# Effectiveness of enhanced filtration interventions for improving indoor air quality in child-serving facilities

**DOI:** 10.3389/fped.2026.1875263

**Published:** 2026-08-04

**Authors:** Tian Xia, Julia Raneses, Morgan Locke, Raquel Garcia, Alison Walding, Ricardo F. DeMajo, Amy J. Schulz, Stuart A. Batterman

**Affiliations:** 1 Environmental Health Sciences, School of Public Health, University of Michigan, Ann Arbor, MI, United States; 2 Health Behavior and Health Equity, School of Public Health, University of Michigan, Ann Arbor, MI, United States; 3 Southwest Detroit Environmental Vision, Detroit, MI, United States

**Keywords:** environmental justice (EJ), indoor air quality (IAQ), particulate matter (PM), portable air cleaner (PAC), child-serving facilities, teacher behavior

## Abstract

Indoor air quality (IAQ) in schools and other child-serving facilities is crucial for the health and development of children, a large and vulnerable population. Interventions to improve IAQ, e.g., enhancing HVAC system filtration and deploying portable air cleaners (PACs), are especially needed at facilities located in polluted areas. Evidence of the actual effectiveness of these interventions, however, is limited and the reported performance is inconsistent. The objectives of this study, a component of a larger community-based participatory research partnership, were to evaluate the effectiveness of, and factors influencing, interventions using enhanced filtration; compare standard school buildings with other child-serving facilities such as nurseries, community centers, and childcare houses; and to identify IAQ improvement challenges and suggest relatively low-cost and effective mitigation methods. We evaluated 33 spaces in 12 facilities that served children from daycare through high school ages. Week-long baseline and follow-up measurements and teacher/staff surveys were obtained before and after the filter interventions. In most facilities, thermal comfort was acceptable, but ventilation rates were low (air change rates from 0.9–1.4 h^−1^). At baseline during school hours, indoor levels of PM_2.5_ averaged 2.2–6.3 μg/m^3^ and indoor PM_10_ averaged 5.7–26.9 μg/m^3^. Standard schools saw the highest particulate matter (PM) levels. The intervention reduced PM_2.5_ and PM_10_ concentrations in the standard schools and nursery (by 45 to 54% for PM_2.5_), but had little effect at childcare houses. A linear mixed-effects model (LMM) indicated a ∼51% reduction of indoor PM_2.5_ levels by the PACs. For standard school classrooms and the nursery, the effectiveness of filtration strategies could be predicted using single-zone mass balance models, but predictions were inconsistent for portable buildings, facilities containing very large rooms, and houses repurposed for childcare. Overall, enhanced filtration can improve IAQ in child-serving facilities, but the long-term effectiveness, energy and economic costs require consideration. We recommend that teachers and staff acquire appropriate training and education to support and sustain effective and cost-efficient IAQ improvements.

## 1 Introduction

Indoor air quality (IAQ) remains a continuing health concern due to its association with allergies, asthma, cancer, cognitive dysfunction, attention span, and mortality (1–7). IAQ in child-serving facilities, which include schools, nurseries, day care, and after school facilities, is crucial since children are susceptible to adverse effects of indoor air pollutants, and they spend considerable time in these settings (2, 8–15). Exposure to fine particulate matter (PM_2.5_), for example, has been associated with respiratory symptoms, asthma, cancer and mortality (1, 7, 9, 10, 16). PM_2.5_ containing pathogens can promote transmission of infectious diseases, such as influenza and SARS-CoV-2 (17, 18). Wildfire smoke events can worsen both outdoor and indoor PM_2.5_ pollution, posing additional health threats (19–22). Elevated carbon dioxide (CO_2_) levels, another concern in schools, has been associated with decrements in student school performance (3, 23) and attendance (24). CO_2_ measurements also are used to estimate classroom ventilation and air change rates (ACR) (25–27).

Many studies have measured IAQ parameters in traditional schools, e.g., typically buildings with multiple classrooms along large corridors, however, few studies have examined IAQ in other types of child-serving facilities, e.g., nurseries, community centers, and childcare provided in repurposed residences (subsequently called childcare houses). Such facilities often serve preschool children (under six years old), who are exceptionally vulnerable to pollutants due to their immature immune and respiratory systems. A recent review of nurseries in 12 countries (28) reported that examined classrooms generally meet comfort (temperature and humidity) guidelines (29), but CO_2_ levels frequently exceeded 1,000 ppm due to deficient ventilation, and PM_2.5_ and PM_10_ concentrations frequently exceeded the WHO guidelines (30). The review suggested that results were highly location dependent, however, the single U.S. study included did not measure indoor PM levels. Other studies have shown that traffic-related emissions (31, 32) and wildfire smoke events (21, 22) can increase indoor PM_2.5_ levels in preschool and childcare facilities. Another type of child-serving facility, residences that have been repurposed for childcare and after school programs, has less frequently been addressed in the intervention literature. A number of studies examining residences with children have reported high PM and CO_2_ concentrations (33–35). IAQ in child-serving repurposed houses may differ from residential homes, a result of more occupants, different indoor activities, and different cleaning and ventilation schedules. Such facilities are common, e.g., the case study city of Detroit has 291 child serving facilities in repurposed houses (36).

Ventilation and filtration are the major methods used to improve IAQ (37, 38). While ventilation serves to dilute and expel the indoor pollutants generated from building sources and occupants including CO_2_ (39), it can simultaneously promote the penetration of outdoor pollutants such as PM_2.5_ (40). Filtration can be achieved by installing filters in mechanical ventilation systems or by using portable air cleaners (PACs) in rooms, the latter typically using high-efficiency particulate arresting (HEPA) filters that can help control infectious disease transmission (41). The effectiveness of filtration has been evaluated using field measurements (42–47) and computational modeling (48–50). Studies evaluating its effectiveness in child-serving facilities, however, have yielded inconsistent results. For example, a California, USA study in a standard school found no clear correlation between PAC use and indoor PM_2.5_ concentrations, possibly because outdoor concentrations were low and relatively high efficiency filters (Minimum Efficiency Reporting Value MERV-13) were already in use (51). A study at nurseries in London, England reported that window opening increased indoor PM_2.5_ concentrations and thus reduced the observed effectiveness of PACs (40). Another study in three urban elementary schools in the northeastern United States reported 42%–49% reduction in PM_2.5_ levels (52). A greater number of studies have evaluated PAC effectiveness in residences, and reductions of 19%–92% in PM_2.5_ levels have been reported (42, 43, 45, 46, 53). Wildfire smoke events can increase uncertainties in estimating PAC effectiveness (54). In addition to inconsistent effectiveness, filtration requires continuous or extended use of the filter's fans to attain the clean air delivery rate (CADR) required to effectively remove pollutants (55, 56). Filters may not be feasible or acceptable in classrooms due to the filter size, noise, drafts, power needs, and cost (27). The lifecycle cost of filters, which includes costs for purchase, installation, energy penalty, periodic replacement and maintenance, can pose a significant barrier, particularly in resource-constrained environmental justice (EJ) communities (27, 57). Few studies in child-serving facilities have taken both ventilation (and outdoor pollutant concentration) and filter usage into their analysis. Overall, an improved understanding of how ventilation and usage jointly affect the real-life effectiveness of filters is needed. Filter use strategies should be optimized to reduce energy and economic burdens while still achieving IAQ improvements (58).

Child-serving facilities in underserved or EJ communities are often disproportionally threatened by poor IAQ and the associated adverse health effects. In cities like Detroit, Michigan, USA, these communities frequently are near air pollution sources and are exposed to high ambient pollutant concentrations (59–67). Further, many child-serving facilities have outdated infrastructure, inefficient and poorly maintained heating, ventilation, and air conditioning (HVAC) systems, and lack appropriate – or any – air cleaning equipment (43, 68). These factors can promote the penetration of outdoor pollutants into the building, e.g., traffic emissions that can elevate PM_2.5_ and black carbon (BC) concentrations in schools (27).

**Figure 1:**
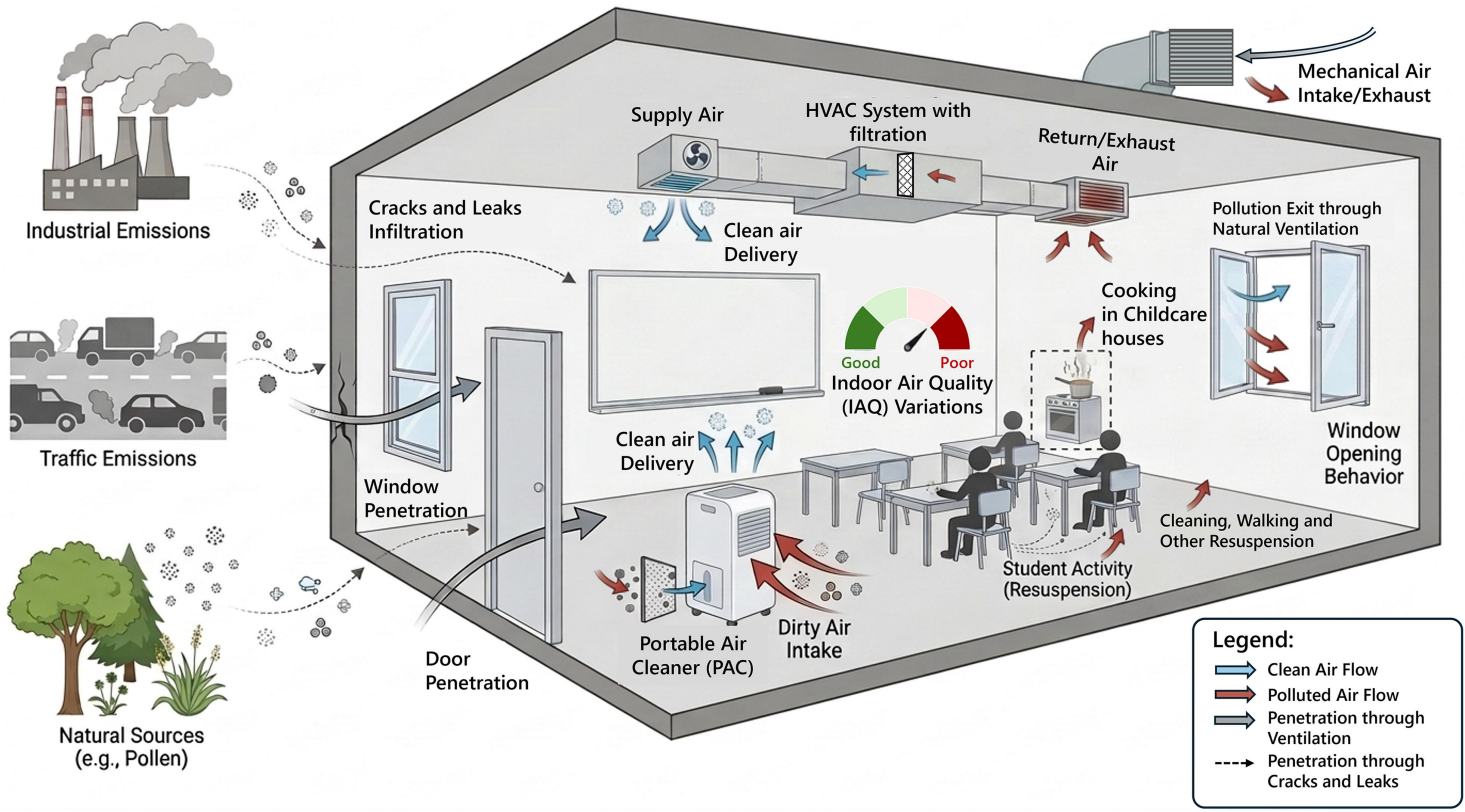
Conceptual figure illustrating how outdoor air pollution, indoor sources, mechanical and natural ventilation, PAC and HVAC filtration, and occupants’ behavior may impact IAQ in schools. The image was partially generated by FigureLabs genAI.

The present study has three objectives: (1) evaluate the effectiveness of, and factors influencing, an intervention using enhanced filtration; (2) compare standard school buildings with other child-serving facilities such as nurseries, community centers, and childcare houses; and (3) identify IAQ improvement challenges and suggest relatively low-cost and effective mitigation methods. These objectives align with recently expressed research priorities, including developing “guidance for IAQ related building assessments”, understanding “the impact of building-level variables” on IAQ outcomes, and determining “low-cost, high impact IAQ improvements for schools” (69). This study monitored IAQ at four types of child-serving facilities (standard school, nursery, community center and childcare house) in EJ communities in Detroit before and after an intervention aimed at enhancing filtration. The monitoring data, together with teacher/staff behavior surveys, provide novel insights on real-world intervention challenges and facility type comparison. Figure 1 conceptualizes the factors affecting IAQ addressed in this study, including outdoor pollution penetration, mechanical and natural ventilation, PAC application, and occupant’ behavior. Indoor sources, e.g., cooking and dust resuspension, were not measured in this study but will be discussed.

## 2 Methods

We report on the IAQ monitoring and interventions at 12 child-serving facilities in the Detroit, Michigan area, a part of the Schools Indoor Environments Project (https://caphedetroit.sph.umich.edu/information-air-quality/schools-indoor-environment-project/) developed under the Community Action to Promote Health Environment (CAPHE) partnership. Details of this community-based participatory research project and the CAPHE partnership can be found elsewhere (27).

### 2.1 Child-serving facilities and study approach

Details of the child-serving facilities are listed in Table 1. In the CAPHE partnership, child-serving facilities in underserved or EJ communities were defined as schools or child-serving facilities located in polluted areas. These include areas adjacent to busy roads or industry with high levels of air pollutants as assessed by the Michigan EJ screen cumulative burden index (a science-based ranking score that identifies Michigan communities disproportionately impacted by combined environmental hazards and socioeconomic vulnerabilities) (70). Most of the participating facilities are located within underserved and EJ communities, and most are near industry, major roads with extensive heavy-duty truck traffic, and large scale construction activities, including the construction of a new international bridge to Canada (71). Industrial and traffic emissions in Detroit result in the highest PM_2.5_ level in the state, and current design values exceed the annual average National Ambient Air Quality Standards (9 μg/m^3^) (72). Mobile emissions cause a large and growing share of Detroit's ambient PM_2.5_ pollution (73–75). The 12 facilities are categorized into four types. Facilities F1 – F4 are classified as “standard” schools and serve students from kindergarten through eighth grade in multiple, similar and dedicated classrooms, each with 15 to 25 students. F1, F2 and F4 are one- and two-story permanent buildings; F3 is a single-story portable building. The other eight facilities are grouped as follows. Facility F5 is a nursery with five dedicated rooms serving infants to preschool children, grouped by age. The three examined large multipurpose community centers (F6, F7 and F12) contain a few rooms for teaching and childcare, in addition to offices, kitchen, eating areas and other spaces. F6 is a single-story education center for after-school (usually 3–7 p.m.) activities such as robotic labs and 3D printing projects for kindergarten through high school students. F7 is a single-story community center that provides preschool to high school childcare services in two dedicated rooms (we examined both) during summer. F12 is a two-story building with basement and first-floor rooms for preschool childcare. Facilities F8 – F11 are former single-family residences converted to preschool childcare facilities and after-school programs (childcare houses). At these facilities, former living rooms, basements, and bedrooms are used as classrooms, nursing rooms, and playrooms, while kitchens and dining rooms have retained their original functions. All facilities are unoccupied at night.

**Table 1.** List of participating child-serving facilities, facility types, ventilation methods, number of inspected rooms, HVAC system, intervention procedure and monitoring months. Additional information, including average room sizes and PAC placement, can be found in Table S5 in the supplemental information (SI). MERV: Minimum Efficiency Reporting Value; N: naturally ventilated; M: mechanically ventilated; CAV: constant volume air handling unit; AHU: Air handling unites.

ID	Facility Type	Venti-lation	# of Rooms	Heating	Cooling	Baseline Treatment	Intervention^1^	Baseline Month	Follow-up Month
F1	Standard School	N	3	Radiator	Window AC	1 PAC	2 PACs in each classroom, mostly Whispure^8^^,^^9^	Mar	Jun
F2	Standard School	M	3	AHUs^2^	AHUs	1 PAC + MERV 7 (poorly installed)	Upgrade to MERV13^9^	Apr	Jun
F3	Standard School	M	2	AHUs^3^	AHUs	1 PAC + MERV 7	Upgrade to MERV13^9^	Apr	May
F4	Standard School	N	3	Radiator	Window AC	Unused DIY Filtration	2 PACs in each classroom^8^	Jan	Apr
F5	Nursery	N & M	3	Radiant floor	Forced air	1 PAC	Filter replacement,^10^ consistent usage	Apr	May
F6	Community center	M	3	AHUs^4^	AHUs	MERV8	NA	Jul	NA
F7	Community center	M	3	CAV^5^ and AHUs^6^	AHUs	MERV10	NA	Aug	NA
F8	Childcare house	M	3	Forced air^7^	Window AC	MERV11 + Unused PAC	Upgrade to MERV13	Feb	Apr
F9	Childcare house	M	3	Forced air^7^	Forced air	MERV10 + Unused PAC	Upgrade to MERV13	Feb	Apr
F10	Childcare house	M	3	Forced air^7^	Forced air	MERV10 + Unused PAC	Upgrade to MERV13	Feb	May
F11	Childcare house	N	2	Radiator	Window AC	Unused PAC	Filter replacement,^11^ consistent usage	May	Jul
F12	Community center	N	2	Forced air	Window AC	Unused PAC	Filter replacement,^11^ consistent usage	Jun	Aug

1 The facilities were generally provided with PACs and HVAC filter replacements for a year. The staff were instructed to replace the HVAC filters every three months, and PAC filters annually or as suggested by the manufacturer. The teachers and staff were instructed to operate PAC at the highest setting they could tolerate without noise issues during school hours and run the PAC during unoccupied hours if they prefer.

2 3 AHUs: 1 manufactured in 1970s, 4,000 ft^3^/min, 2 manufactured in 2010, 6,000 ft^3^/min. All used MERV 7 filters with 3-month replacements. The filters were often not properly installed, resulting in folds and/or damage. All set at auto fan speed (no ventilation unless there is a cooling/heating need).

3 4 AHUs: one for each room in the portable, all manufactured in 2015, 1,500 ft^3^/min. All used MERV 7 filters with 3-month replacements. All set at auto fan speed.

4 5 AHUs and 3 additional AC units. For the AHUs: 1 made in 2018, 2,400 ft^3^/min; 1 made in 2015, 3,000 ft^3^/min; 2 made in 2018, 4,000 ft^3^/min; 1 made in 2018, 5,000 ft^3^/min. All used MERV 8 filters with 3-month replacements. All set at auto fan speed.

5 The older part of the building was mechanically ventilated by the original CAV installed in 1960s. Most dampers were missing or not functioning. Closed dampers led to no outdoor air intake. MERV 10 filters were installed while some slots saw missing or not properly installed filters. The filter replacement schedule was unknown, but the inspection indicated no replacement for a long time. The fan kept running throughout the day.

6 The newer part of the building had 4 AHUs installed on the roof. All manufactured in 2012 – 2013. MERV 10 filters were installed with routine replacement by a maintenance company. The fan kept running throughout the day.

7 The three childcare houses have conventional central forced-air HVAC systems. All set at auto fan speed. Filter replacements were infrequent. Reverse installation, wrong size or folded filter were frequently observed.

8 Whirlpool WP500 Whispure HEPA Air Purifier, HEPA filtration, 4-speed, measured maximum CADR: 404 ft^3^/min (686 m^3^/hr).

9 Medify MA-40 Air Purifier, HEPA filtration, 3-speed, rated maximum CADR: 239 ft^3^/min (406 m³/h).

10 Medify MA-50 Air Purifier, HEPA filtration, 4-speed, measured maximum CADR: 340 ft^3^/min (578 m³/h).

11 Crane EE-5068 Air Purifier, HEPA filtration, 3-speed, measured maximum CADR: 39 ft^3^/min (66 m^3^/hr).

The study approach, detailed elsewhere (27), included: 1) prioritization and selection of facilities guided by community partner organizations involved with CAPHE as well as the eligibility criteria specified above; 2) an initial inspection to document building characteristics and to select locations for environmental monitoring (in two or three rooms and an outdoor location, if an outdoor electrical outlet was available); 3) subsequent visits to conduct continuous monitoring for at least one week to establish “baseline” conditions; 4) visits to deploy enhanced filters; 5) visits for “follow-up” monitoring, again for at least 1 week; and 6) reporting back to facility personnel. At the 12 facilities, monitoring was conducted in 33 rooms. Baseline and follow-up measurements separated by an average of 2.1 months (ranged 1–3 months) (Table 1). As described below, interventions were tailored to each space, and teachers and staff throughout the facility were surveyed before and after the intervention.

### 2.2 IAQ monitoring instrumentation

IAQ measurements were collected by seven instruments integrated into a custom-built case. Carbon dioxide (CO_2_) was monitored (C7632A, Honeywell, Charlotte, NC, USA) and used to estimate air change rates (ACR). Temperature and relative humidity (T, RH) was monitored using an indoor environment sensor (HOBO U10-003, Onset, Bourne, MA, USA). A 5-channel optical particle counter (OPC; Aerocet 531S, Met One, Grants Pass, OR, USA) measured PM_1_, PM_2.5_, PM_4_, PM_7_, PM_10_, and TSP. A portable aethalometer measured black carbon (BC; MA200, AethLabs, San Francisco, CA, USA). A sound pressure level (SPL) meter (SD-4023, Reed Instrument, Wilmington, NC, USA) measured indoor noise levels. Indoor air was pumped through an inlet at the child's breathing height (1 m) to the instruments; the SPL microphone was installed at 1 m height as well. A second CO_2_ sensor with a higher range (5,000 ppm; HOBO MX1102A, Onset, Bourne, MA, USA) was installed in rooms with high CO_2_ levels (>2000 ppm). Due to availability constraints, a second PM and BC instrument (ObservAir, DSTech, Berkeley, CA, USA) was used at F4. The placement of monitors was primarily determined by the room setup and the following rules: 1) clear of obstacles to air flow; 2) not interfering with student activities; 3) access to a wall power outlet; 4) if possible, away from windows and doors; and 5) if possible, away from PACs, ACs and air vents. The same system was used for baseline and follow-up monitoring in the same room to reduce measurement uncertainty. Photos of the instrument and the instrument setup are shown as Supplementary Figure S6 in the SI.

Instrument specifications, data validation and consolidation, quality assurance (QA), and instrument calibration procedures have been detailed elsewhere (27). In brief, all instruments were collocated and calibrated before sampling at each facility or every six months. CO_2_ sensors were calibrated using a certified gas standard (1,010 ppm), SPL meters were calibrated using a 94 dBA calibrator, and other instruments were collocated and calibrated to the measurement average. PM_2.5_ measurement errors after calibration were ∼3%, PM_10_ measurement errors after calibration were within 10%. BC measurements errors ranged from 20 to 30%.

Simultaneous outdoor air measurements were collected at each facility using the same set of instrumentation noted above if a suitable electrical outlet was available. The lack of an outlet, staff unplugging the sampling system, or instrument failure limited outdoor measurements at five facilities (F1, F2, F3, F10, F12). Ambient conditions at these facilities were estimated by averaging hourly PM_2.5_, PM_10_, BC, T, and RH measurements at five nearby air quality monitoring sites (noted as SW sites) operated by the State of Michigan.

### 2.3 Teacher survey

Baseline and follow-up surveys [detailed elsewhere (27)] were administered to a total of 100 teachers and staff at the 12 facilities to collect thermal comfort perceptions, information related to IAQ and purifier use, health symptoms potentially related to IAQ, and demographic information (age, sex, smoking status). The survey questionnaire is included in the SI. Here we focus on survey questions related to IAQ and health outcomes: room cleanliness; satisfaction with thermal comfort and indoor air quality; overall IAQ satisfaction; recent symptoms; and perceptions of whether IAQ interferes with or enhances learning. The sample size does not permit statistical testing for the survey measures, especially between facility types. Instead, we tabulate overall numbers and percentages of respondents and qualitatively discuss baseline and follow-up responses. Participants provided informed consent, and all study procedures were approved by the University of Michigan Institutional Review Board (IRB) dated 12/01/2021 and amended on 10/17/2024.

### 2.4 Interventions

Table 1 describes baseline filtration (before the intervention) and the intervention strategy at the 12 facilities. The intervention goal was to attain an effective clean air change rate (ACR plus clean air change per hour due to PAC usage) of 3 to 6 h^−1^ in each room, following ASHRAE guidance (29). Different intervention methods were applied at different facilities, according to room sizes, ventilation system, and budget constraints. The details of and rationale for intervention at each facility are presented in Table 1 and the SI. In brief, at facilities without central ventilation (F1, F4, F5, F11 and F12) and other facilities that had PACs already installed (F2 and F3), 1–3 PACs were installed or serviced (filter replacement and usage monitoring) in each room. At the mechanically ventilated facilities (F2, F3, F8, F9, F10), the intervention upgraded HVAC filters to MERV 13. At two other facilities (F6 and F7), interventions and follow-up measurements were not conducted, due to the infrequent use of the child-serving rooms or large room sizes (400–1,000 m^3^ volume). Large room sizes can lead to significant IAQ measurement uncertainties and filter or PAC purchases that exceeded our budget.

### 2.5 Data analysis

The collected data was cleaned and processed with standardized methods and templates (27). Sampling rates depended on the measurement: CO_2_, T, and RH were measured at 5 min intervals; PM was sampled as 1-min integrated measurements for every 5 min; MA200 collected 5-min integrated BC samples; noise levels in dBA were measured at 1-s intervals and logarithmically averaged in 5-min periods; and the DST sampled BC at 2-s intervals and was averaged over 5-min periods. Then 15-min averages of all 5-min IAQ measurements were calculated and consolidated. The power consumption of each PAC at each fan speed was measured, which was then applied to convert the smart plug power consumption measurements (taken every 10 min) to fan speed and the corresponding CADR. Fan speeds were further classified as off, low (Speeds 1 and 2), and high (Speeds 3 and if applicable, Speed 4).

The consolidated 15-min data for each room was analyzed after excluding measurements outside the 0.5th to 99.5th percentile of the overall indoor or outdoor data to avoid outliers. The screened indoor PM_2.5_ concentrations ranged from 0.1 to 25.4 μg/m^3^, and outdoor PM_2.5_ from 0.3–54.4 μg/m^3^. Start and end times for facility occupancy (occupied periods) were determined from teacher/staff surveys and by noting CO_2_ elevations; generally, the latter method better reflected daily variations based on our observations. Descriptive statistics for IAQ parameters were calculated for occupied and unoccupied periods separately. PM_2.5_ I/O ratios were calculated for each room using daily averages of indoor and outdoor PM_2.5_ levels. I/O ratios normalize indoor PM concentrations against the corresponding outdoor levels, allowing for comparisons of indoor particle infiltration and intervention effectiveness between facilities. The I/O ratio is a common indicator to reflect the effectiveness of ventilation and IAQ improvement strategies (76–79). ACRs were estimated using the CO_2_ decay method, which has been illustrated in detail in our previous publications (27, 80). These parameters were estimated for each room and each day for both occupied and unoccupied conditions. The daily I/O ratios and ACRs were compared between facility types and occupancy conditions, and were used to develop and validate the singe zone IAQ model.

### 2.6 Single zone IAQ model

Steady-state single zone models were used to calculate the expected PM_2.5_ I/O ratios for baseline and follow-up periods in the 33 rooms monitored (detailed equations and parameters are documented in the SI). The model accounts for classroom volume, ventilation, particle deposition to the floor, walls and ceiling, and the PAC usage and the corresponding CADR (if applicable). The model makes several assumptions: a single zone (room), a single particle size (2.5 μm), steady-state conditions, a friction velocity of 0.3 m/s (81), a 0.75 mixing factor for both PAC operation (k_PAC_) and particle deposition (k_D_), and 100% and 50% of the untreated outdoor air in the total air change (p_n_) for naturally and mechanically ventilated facilities respectively. The total air change rate, estimated using the CO_2_ decay, incorporates all mechanisms (ventilation, air infiltration/exfiltration, and building leakage). We assumed no indoor PM_2.5_ generation, which may not reflect conditions in buildings with dirty floors, cooking, or other sources, and can lead to model-measurements disagreements.

### 2.7 Linear mixed-effects model for indoor PM_2.5_

A linear mixed-effects model (LMM) was developed to estimate intervention effectiveness and evaluate factors influencing indoor PM_2.5_ concentrations while accounting for potential confounders and the repeated, clustered structure of the data. The primary LMM included one dependent variable [the natural log of indoor PM_2.5_ concentration, ln(PM_2.5,in_)], 12 fixed effect variables, and one random effect variable, monitored rooms nested within schools, to represent repeated measurements and hierarchical clustering. The fixed effect variables included five methodological parameters [absolute indoor-outdoor temperature difference, outdoor RH, wind speed, sin(wind direction), cos(wind direction)], four room parameters (volume, ACR, ventilation type and occupancy), two categorical intervention parameters (filter upgrade and PAC added) indicating the intervention status, and the outdoor PM_2.5_ concentration. Outdoor PM_2.5_ was included to represent ambient pollution conditions and the impacts of PM penetration. Occupancy was an indicator of indoor activities that may generate or resuspend PM. Temperature difference, outdoor RH, wind speed and direction were included to represent meteorological conditions that may affect outdoor pollution levels, indoor-outdoor air change rates, and occupant ventilation behavior. ACR, room volume, and ventilation type accounted for building and ventilation characteristics. Indoor and outdoor PM_2.5_ levels were natural log transformed to reduce skewness. Occupancy was modeled as ln(occupancy+1) to account for periods when rooms were unoccupied. Ventilation type was a categorical parameter (1 = naturally ventilated, 0 = mechanically ventilated). For intervention, filter upgrade was coded as 1 for mechanically ventilated rooms after filter upgrades and extended fan operation, and 0 otherwise. PAC added was coded as 1 for naturally ventilated rooms after PAC installation or service, and 0 otherwise. The model equation is:

$$\ln\;(PM_{2.5,\mathrm{in},\mathrm{ijk}})=\;\beta_{0}+\;\overset{12}{\underset{m\;=\;1}{\sum }}\;\beta_{m}\;X_{m,\mathrm{ijk}}+\;b_{\mathrm{ij}}+\;\varepsilon_{\mathrm{ijk}}$$ (1)

where i = school index, j = room index, k = observation index, m = index for the fixed effect variables, PM_2.5,in_ = indoor PM_2.5_ concentration, *β* = model estimated fixed effect parameters, X = fixed effect variables, b = random intercept, and *ε* = residual error. After running the full model, insignificant variables were excluded and the final model used significant covariates (both intervention factors were included). We also modeled PM_2.5_ I/O ratios and outdoor PM_2.5_ (detailed in Supplementary Tables S10 and S11*)*.

### 2.8 Energy consumption and cost analysis

Energy consumption and cost analyses were conducted for three scenarios based on field study cases: mechanically ventilated standard classrooms with enhanced HVAC filtration, naturally ventilated standard classrooms with PACs, and childcare houses with enhanced forced-air system filtration. Current prices of filters and PACs were used; inflation was not considered. Assumptions, methods, and additional data are given in Supplementary Table S8 and a previous publication (50). In brief, for mechanically ventilated standard classrooms, we assumed a 12.5-ton rooftop AHU with 6 pleated (18  ×  24 × 2 inches) MERV-8 filters that served 4 standard classrooms, each with 25 students; the enhanced filtration intervention involved upgrading the filters to MERV13, seasonal filter replacement, and keeping the supply fan running throughout the school hours. The ACR was unchanged, thus the heating and cooling load was unchanged. For the naturally ventilated standard classroom with 25 students, we assumed that the intervention installed two PACs (Whirlpool WP500 Whispure HEPA Air Purifier), which were operated at the highest fan speed throughout the school day. Each PAC was assumed to last for ten years, and the annualized capital cost was 1/10 of the PAC price. For childcare care houses, we assumed that each house served 15 children, upgrading the forced-air HVAC system filter (16 × 24 × 1 inches) from MERV8 to MERV13, quarterly filter replacement, and continuous use during school hours. Again, the heating and cooling load was assumed unchanged. Results were expressed as the change in the annual energy consumption per classroom/facility, and the total cost differential, which included costs of filters, energy, and the PACs. Additionally, the total cost was expressed per year per student.

### 2.9 Sensitivity analysis

The single zone model was optimized to increase agreement with PM_2.5_ measurements at the standard schools (F1 – F4; excluding one outlier as discussed in Section 3.5) using sensitivity analyses. We evaluated different data screening criteria (e.g., lower and upper limits to avoid outliers) and the representativeness of outdoor PM_2.5_ concentration estimations (e.g., nearby air quality monitoring sites or local measurements by our instruments). We also evaluated parameters in the single zone model, including the percentage of untreated outdoor air in the total air change (p_n_), and mixing factor for PAC operation (k_PAC_) and particle deposition (k_D_). All sensitivity analysis results are documented in the SI.

Data processing, statistical analysis, modeling, and results visualization work were completed using Microsoft Excel and Origin(Pro), Version 2026 (OriginLab Corporation, Northampton, MA, USA).

## 3 Results

Unless otherwise indicated, the results discussed in this section focus on measurements collected during the occupied periods. Data from unoccupied periods are presented but are not discussed comprehensively.

### 3.1 IAQ overview

IAQ measurements are summarized in Table 2, and the corresponding outdoor data are summarized in Supplementary Table S6 in the SI. At baseline, the standard schools had higher PM_2.5_, PM_10_ and CO_2_ levels, but lower BC levels than the other childcare facilities (nursery, community center and childcare houses, *p* < 0.05 except for BC in nursery). Indoor temperatures averaged 21.5–26.7 °C and RHs averaged 22.6–47.9%. Indoor temperature generally fell within the EPA guidelines (19.7–26.7 °C) (82) and Michigan guidelines for child care centers (>20 °C) (83) with two exceptions (one room in F5 was slightly warm, 27.1 °C, and one room in F9 was too cool, 17.8 °C). RH typically fell below guidelines (30%–60%) (82) in one standard school (F4, 25%–30%), the nursery (F5, 24%–27%) and in most childcare houses (F8, F9 and F10, 15%–21%). Childcare houses generally had the most variable temperatures and RH, likely due to infiltration and teacher/staff behavior (e.g., opening/closing windows and window AC usage).

**Table 2:** Summary and comparison of six IAQ parameters for four facility categories during occupied and unoccupied periods during the baseline and follow-up sampling. Baseline nursery, community center and childcare house measurements were compared with school measurements. Follow-up measurements were compared with the corresponding baseline.

	Standard School		Nursery		Community Center		Childcare Houses	
Parameter	Average (SD)	N	Average (SD)	N	Average (SD)	N	Average (SD)	N
A) Summary of IAQ measurements during occupied periods								
*Baseline*								
PM_2.5_ (*μ*g/m^3^)	6.30 (3.68) ^a^	42	2.20 (1.30) **	15	3.91 (2.80) **	44	3.28 (2.23) **	48
PM_10_ (μg/m^3^)	26.85 (13.24) ^a^	42	6.70 (2.17) **	15	5.71 (4.35) **	44	7.36 (4.86) **	48
BC (μg/m^3^)	0.78 (0.48) ^a^	39	1.17 (0.79)	15	1.17 (0.87) *	43	1.04 (0.51) *	52
CO_2_ (ppm)	1484 (394) ^a^	47	797 (114) **	15	891 (145) **	44	705 (155) **	48
T ( °C)	22.8 (0.8) ^a^	47	26.7 (1.3) **	15	23.4 (1.2) *	44	21.5 (2.1) **	52
RH (%)	32.3 (5.7) ^a^	47	25.3 (5.5) **	15	47.9 (6.0) **	44	22.6 (9.2) **	52
*Follow-up*								
PM_2.5_ (μg/m^3^)	3.45 (1.97) **	44	1.01 (0.27) **	12	10.70 (2.83)^**^	8	3.11 (2.12)	54
PM_10_ (μg/m^3^)	17.66 (9.33) **	44	2.11 (0.84)^**^	12	17.41 (8.22)^**^	8	6.96 (4.87)	54
BC (μg/m^3^)	0.31 (0.16) **	32	0.72 (0.41)	12	2.03 (0.76)^*^	8	1.01 (0.46)	55
CO_2_ (ppm)	1502 (388)	47	750 (84)	12	1138 (289)^*^	8	785 (171)^*^	55
T ( °C)	23.2 (1.3)^*^	47	24.6 (1.1)^**^	12	22.8 (0.8)	8	22.3 (2.6)	55
RH (%)	40.3 (10.8) **	47	34.0 (3.8)^**^	12	40.2 (2.6)^**^	8	33.7 (10.2)^**^	55
B) Summary of IAQ measurements during unoccupied periods.								
*Baseline*								
PM_2.5_ (μg/m^3^)	3.75 (2.83) ^a^	64	1.10 (0.68)^**^	21	3.29 (2.21)	56	1.10 (0.97)^**^	71
PM_10_ (μg/m^3^)	8.05 (6.58) ^a^	64	1.54 (1.06)^**^	21	3.99 (2.90)^**^	56	1.51 (1.47)^**^	71
BC (μg/m^3^)	0.50 (0.27) ^a^	62	0.90 (0.57)^**^	21	1.23 (0.82)^**^	54	0.62 (0.31)^*^	77
CO_2_ (ppm)	712 (212) ^a^	75	521 (55)^**^	21	652 (99)^*^	56	543 (118)^**^	70
T ( °C)	20.8 (1.5) ^a^	75	26.6 (0.9)^**^	21	23.2 (1.2)^**^	56	20.8 (2.1)	77
RH (%)	28.3 (5.8) ^a^	75	20.1 (3.9)^**^	21	47.3 (6.3)^**^	56	21.0 (8.4)^**^	77
*Follow-up*								
PM_2.5_ (μg/m^3^)	1.56 (0.94)^**^	70	0.78 (0.30)	21	6.85 (3.64)^**^	14	1.42 (1.54)	76
PM_10_ (μg/m^3^)	4.02 (3.13)^**^	69	0.91 (0.32)^*^	21	7.92 (3.79)^**^	14	1.98 (2.55)	76
BC (μg/m^3^)	0.27 (0.23)^**^	49	0.58 (0.33)^*^	21	1.71 (0.84)	14	0.65 (0.42)	77
CO_2_ (ppm)	605 (130)^**^	74	514 (49)	21	674 (136)	14	563 (160)	77
T ( °C)	22.9 (1.5)^**^	74	23.8 (0.8)^**^	21	22.7 (0.7)^*^	14	21.8 (3.3)^*^	77
RH (%)	41.0 (9.2)^**^	74	31.8 (3.8)^**^	21	41.7 (3.2)^**^	14	32.8 (10.3)^**^	77

* *p*-value < 0.05; **, *p*-value < 0.01; two-sample t-test.

a Not compared with other data.

PM concentrations at baseline decreased during unoccupied hours (compared to occupied periods), e.g., standard schools and nurseries had substantial PM_2.5_ and PM_10_ reductions (40 to 77%); BC reductions were somewhat lower (23 to 36%). Childcare houses saw slightly larger reductions. Levels at community centers showed modest differences. Again, the standard schools had higher PM_2.5_ and PM_10_ levels but lower BC levels than the other categories of child-serving facilities. PM_2.5_, PM_10_, and BC levels in nursery, community center and childcare houses differed from measurements in standard schools at baseline (*p*-value < 0.05). CO_2_ concentrations dropped to 521–712 ppm, higher than the ambient level (∼ 420 ppm), indicating low air exchange during unoccupied periods.

After the intervention, standard schools and the nursery saw nearly 50% reductions in average levels of PM_2.5_, PM_10_ and BC compared to baseline, both during occupied and unoccupied periods. Large increases for community centers were seen; however, these results are not representative since only one facility had follow-up measurements. The follow-up measurements at this facility (F12) were impacted by wildfire smoke events (84), which led to elevated outdoor PM_2.5_ concentrations (38.4 ± 13.3 µg/m^3^ during occupied hours; 28.5 ± 13.4 µg/m^3^ during unoccupied hours, Supplementary Table S6 in the SI) while BC levels (0.45 ± 0.05 µg/m^3^ during occupied hours; 0.72 ± 0.37 µg/m^3^ during unoccupied hours) were similar to those observed during non-smoke periods. Results at the childcare houses for the three pollutants were inconsistent. Differences between baseline and follow-up periods are compared using I/O ratios in Section 3.4.

### 3.2 Air purifier usage

PAC usage is summarized in Table 3. At the standard schools, teachers used PACs for 94% and 74% of the occupied and unoccupied times, respectively. PACs used the high-speed settings for 52% of the operation time. In the nursery, usage averaged 50% and 29% of occupied and unoccupied hours. When operating during occupied hours, the PACs were mostly run at high speed (74%). Community centers operated PACs for 72% and 47% of occupied and unoccupied hours, respectively. PACs were set to high speed for 54% of the running time. In the childcare houses, PACs usage averaged 54% of occupied hours but only 18% of unoccupied hours. When operating during occupied hours, the PACs were almost exclusively run at high speed (93%). Overall, PACs were used most consistently in the standard schools and the community center. All facility types were able to maintain >50% of running time during occupied hours with PACs frequently set at high speed. PAC usage during unoccupied hours saw 20% – 36% reduction at all facility types.

**Table 3:** PAC usage for three fan speed categories (off, low speed and high speed). Based on seven facilities (four standard schools, one nursery, one community center, one childcare house). Baseline nursery, community center and childcare house measurements were compared with school measurements. Follow-up measurements were compared with the corresponding baseline.

	Standard School		Nursery		Community Center		Childcare Houses	
Fan speed	Average (SD)	N	Average (SD)	N	Average (SD)	N	Average (SD)	N
*Occupied*								
Off	6% (22%)^a^	59	50% (46%)**	12	28% (45%)*	24	46% (48%)*	11
Low	44% (48%)^a^	59	13% (31%)**	12	33% (48%)	24	4% (10%)**	11
High	49% (49%)^a^	59	37% (41%)	12	39% (49%)	24	50% (47%)	11
*Unoccupied*								
Off	26% (40%)**	96	71% (41%)	21	53% (44%)*	42	82% (30%)*	15
Low	34% (45%)	96	24% (41%)	21	12% (22%)*	42	1% (3%)	15
High	40% (47%)	96	5% (17%)*	21	36% (47%)	42	17% (30%)	15

*: *p*-value < 0.05; **: *p*-value < 0.01; two-sample t-test.

a Not compared with other data; .

### 3.3 ACR comparisons

ACRs ranged from 0.9 to 1.4 h^−1^ with generally no significant differences between baseline and follow-up periods or between facility categories (Table 4). At the standard schools, ACRs were higher than the other three facility categories during school hours (1.4 h^−1^), and lower during unoccupied hours (0.8 h^−1^). At the nursery, ACRs showed similar diurnal variation. At the community centers, ACRs averaged 1.3 h^−1^ and 1.1 h^−1^ at baseline during occupied and unoccupied hours, respectively, and dropped to 1.0 h^−1^ and 0.6 h^−1^ respectively at follow-up. It should be noted that only one community center (F12) had follow-up measurements and the reduced ACRs may reflect the impact of window closing during wildfire smoke events (84). At the childcare houses, ACRs did not show obvious changes between occupied baseline, occupied follow-up and unoccupied baseline periods (0.9–1.1 h^−1^), but dropped to 0.7 h^−1^ during the follow-up unoccupied periods, probably reflecting lower duty cycles of HVAC systems (the system is running less often or for shorter periods while maintaining the desired temperature) in warmer weather (April – July).

**Table 4:** Estimated ACR during occupied and unoccupied periods based on baseline and follow-up CO_2_ measurements. Baseline nursery, community center and childcare house measurements were compared with school measurements. Follow-up measurements were compared with the corresponding baseline.

	Standard School		Nursery		Community Center		Childcare Houses	
Sampling period	Average (SD)	N	Average (SD)	N	Average (SD)	N	Average (SD)	N
*Occupied*								
Baseline	1.4 (0.6)^a^	40	1.3 (0.6)	13	1.3 (0.8)	34	0.9 (0.5) **	37
Follow-up	1.4 (0.6)	41	1.2 (0.7)	11	1.0 (0.5)	7	1.1 (0.8)	37
*Unoccupied*								
Baseline	0.8 (0.4) ^a^	49	0.9 (0.5)	15	1.1 (0.6)^*^	36	1.0 (0.5)^*^	45
Follow-up	0.8 (0.4)	42	0.7 (0.2)	12	0.6 (0.4)^*^	7	0.7 (0.4)^**^	51

*: *p*-value < 0.05; **: *p*-value < 0.01; two-sample t-test.

a Not compared with other data; .

### 3.4 I/O ratios

**Figure 2:**
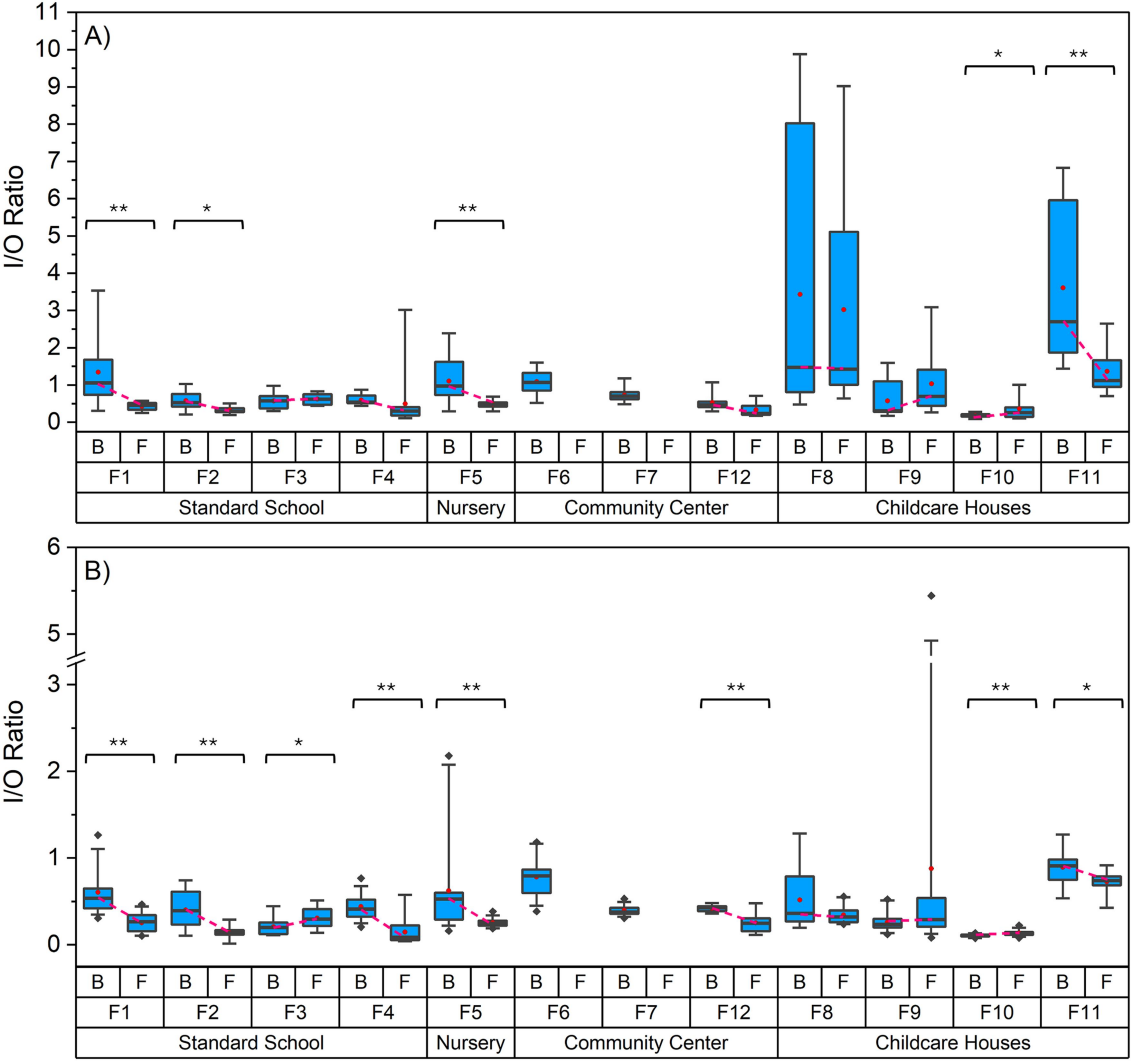
PM_2.5_ I/O ratios at baseline (B) and follow-up (F) at 12 child-serving facilities. **(A)** Occupied period; **(B)** Unoccupied period. Blue boxes represent 25th to 75th percentiles, the middle black line represents median, purple dots represent average values, bars represent 5th to 95th percentiles, and black diamonds represent outliers. *: *p*-value < 0.05; **: *p*-value < 0.01; two-sample t-test.

I/O ratios for PM_2.5_ at baseline and follow-up are shown in Figure 2A. Seven facilities had lower ratios in the follow-up period (F1, F2, F4, F5, F12, F8, F11), as expected due to the filter intervention; changes were statistically significant at four facilities (*p* < 0.05), including three of the four standard schools (F1, F2 and F4) and the nursery (F5). Reductions were very large at F1 and F5, both naturally ventilated permanent buildings with dedicated classrooms. I/O ratios at the portable school building (F3) were unchanged. I/O ratios at the community center (F12) were low and further reduced after intervention (0.53 ± 0.24 to 0.33 ± 0.20). I/O ratios at childcare houses were variable, especially at F8 and F11. I/O ratios at F8 at baseline (3.43 ± 3.63) dropped only slightly at follow-up (3.02 ± 2.88), while F11 did see a drop from baseline (3.60 ± 2.14) to follow-up (1.36 ± 0.64; *p* < 0.01). I/O ratios at F9 and F10 increased following the intervention. These variations could be due to indoor sources, such as re-entrained dust from class activities and indoor emissions from cooking or burning candles (85, 86), which were not fully characterized in this study.

During the unoccupied period, PM_2.5_ I/O ratios (Figure 2B) showed similar changes as during occupied hours, e.g., ratios dropped following the intervention at 7 facilities (F1, F2, F4, F5, F12, F8, F11), of which six were significant (*p* < 0.05). I/O ratios increased at only one non-childcare house facility (F3). Measurements during the unoccupied period did not show the variation seen earlier at F8 and F11, and the community center F12 showed a large reduction, from 0.42 ± 0.04 to 0.25 ± 0.10 (*p* < 0.01).

### 3.5 Single zone model predictions

**Figure 3:**
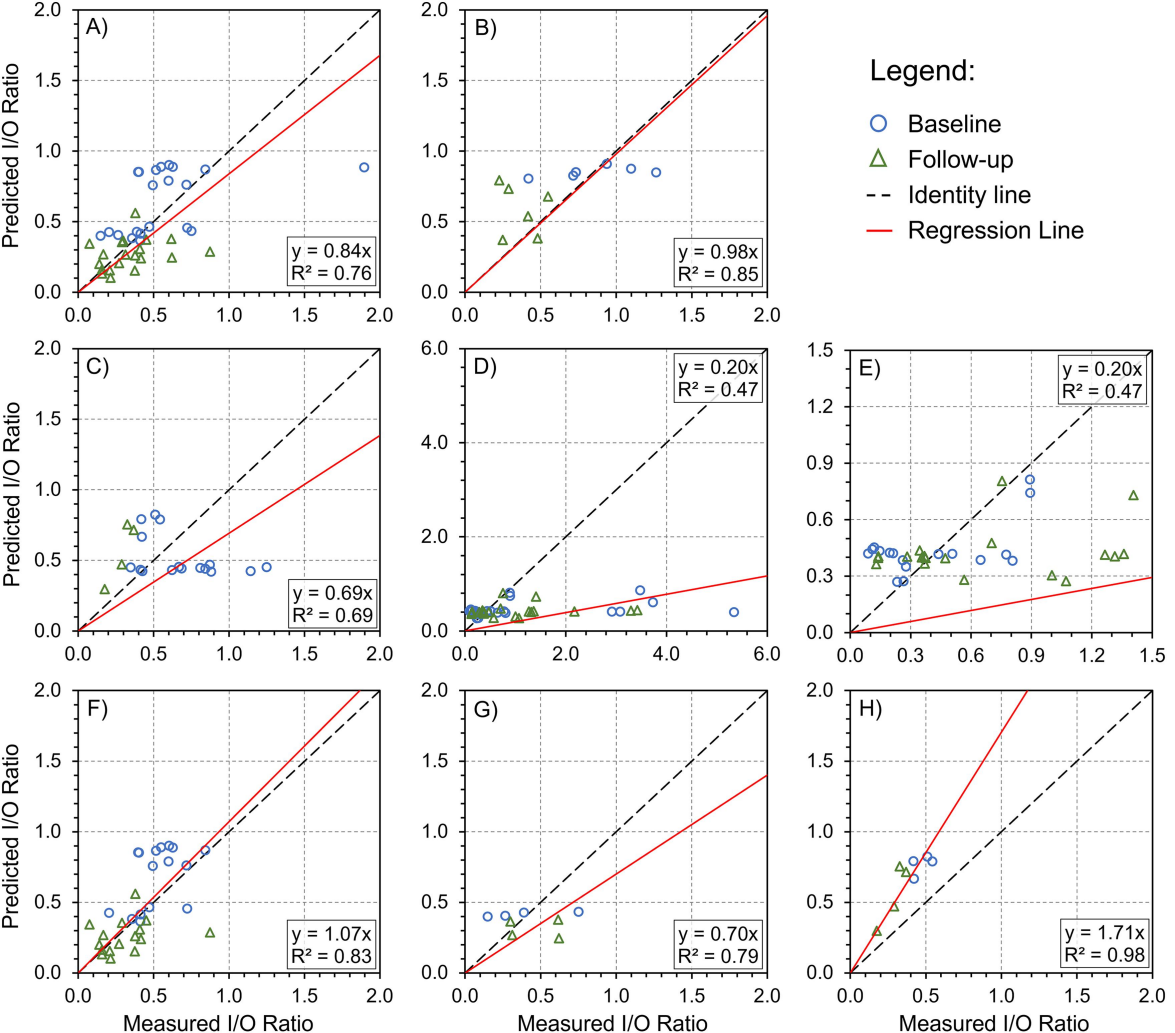
Single zone model estimates of PM_2.5_ I/O ratios vs. measured ratios for **(A)** standard schools; **(B)** nursery; **(C)** community centers; and **(D)** childcare houses. **(E)** is a magnified view of **(D)** between 0 and 1.5 I/O ratios. **(F)** is a plot for all permanent building school facilities (F1, F2 and F4) with one outlier removed. **(G)** plots ratios for the portable standard school building (F3); **(H)** plots ratios for the community center with baseline and follow-up measurements (F12). The dashed black line is the identity line (1-to-1 line). The red regression line was set with zero intercept.

PM_2.5_ I/O ratio predictions using the single zone model provided moderate to strong fits to measurements at most standard schools and the nursery, but not at most community centers or childcare houses (Figure 3). At the standard schools (F1, F2, F3, F4), predicted I/O ratios were slightly low (slope = 0.84) although the fit was acceptable (R^2^ = 0.76; Figure 3A). F3 had the worst fit among the standard schools (Figure 3G). One measurement at F1 at baseline had an unusually high measured I/O ratio (1.90), likely due to an uncharacterized indoor emission event, and was considered an outlier. Removing this observation and F3 data led to strong agreement between predictions and measurements at the standard schools (slope = 1.07, R^2^ = 0.83, Figure 3F). At the nursery, model fit was also strong (slope = 0.98, R^2^ = 0.85; Figure 3B). The model showed good agreement with the measurements at one community center, F12 (slope = 1.71; R^2^ = 0.98; Figure 3H), but generally poor agreement at the other community centers (F6 and F7, Figure 3C) and at all childcare houses (F8, F9, F10, F11; Figures 3D,E).

### 3.6 LMM model results

The final mixed-effects model showed good overall fit, with R = 0.90 and adjusted R^2^ = 0.80, an intraclass correlation coefficient of 0.39, and the *β* values with expected directions (Table 5). The intraclass correlation coefficient indicated that approximately 39% of the variance was attributable to clustering rooms nested within schools, illustrating the impact of facility and building characteristics. Indoor PM_2.5_ was significantly impacted by outdoor PM_2.5_, e.g., doubling outdoor PM_2.5_ concentrations increased indoor PM_2.5_ by 46% (*β* = 0.55, *p*-value = 4.2 × 10^−9^). Addition of PACs was associated with a 51% reduction in indoor PM_2.5_ levels (*β* = −0.72, *p*-value = 5.8 × 10^−7^). Filter upgrades for mechanical ventilation systems led to a 22% reduction though this association was not significant (*β* = −0.24, *p*-value = 0.24), possibly due to the very different systems at different facilities and the uncharacterized variations in fan operation schedule. Many of the covariates were significant. Indoor PM_2.5_ was positively associated with occupancy (*β* = 0.28, *p*-value = 1.1 × 10^×13^), suggesting that PM generation related to indoor activities was significant and should not be omitted. Naturally ventilated rooms saw ∼59% higher indoor PM_2.5_ levels than mechanically ventilated room (*β* = 0.46, *p*-value = 0.0034), potentially due to outdoor PM penetration, limited filtration, or more variable ventilation conditions. The absolute indoor-outdoor temperature differences and outdoor RH were significant meteorological predictors, e.g., 1 °C increase was associated with 3% lower indoor PM_2.5_ (*β* = −0.03, *p*-value = 0.0024), and a 1% increase in outdoor RH was associated with 2% higher indoor PM_2.5_ (*β* = 0.02, *p*-value = 0.0017). Larger temperature differentials can lead to faster air changes, while outdoor PM_2.5_ concentrations were strongly associated with relative humidity (*β* = 0.028, *p*-value = 2.2 × 10^−4^, Supplementary Table S11). The largest temperature differences occurred during heating seasons in Detroit, when facilities generally kept windows closed and reduced outdoor-indoor air changes to save energy. This practice reduced outdoor PM_2.5_ penetration but can increase indoor CO_2_ concentrations. Wind speed, wind direction, air change rate, and classroom volume were not statistically significant (Supplementary Table S9). The LMM for PM_2.5_ I/O ratios was generally similar, although model performance was lower due to the uncertainty in I/O ratios (Supplementary Table S10). Overall, the LMM model showed the importance of facility characteristics, occupancy, ventilation type and outdoor pollution on indoor PM_2.5_ levels, and suggests that filtration interventions, especially using PACs, can be effective.

**Table 5.** The final LMM results for ln(PM_2.5,in_) as the dependent variable, nine fixed effect independent variables, and rooms nested within schools as random effects.

Model Statistics	Value			
Dependent Variable	ln(PM_2.5,in_)			
Number of Points	119			
Degrees of Freedom	110			
Reduced Chi-Square	0.13			
Residual Sum of Squares	14.52			
R Value	0.90			
Adj. R-Square	0.80			
Intraclass Correlation	0.39			
Fixed Effect Parameters	β	Standard Error	t-Value	p-Value
Intercept	-1.62	0.46	-3.55	5.7E-04
Indoor-outdoor absolute temperature difference	-0.03	0.01	-3.11	2.4E-03
Outdoor RH	0.02	0.01	3.22	1.7E-03
Ln(PM_2.5,out_)	0.55	0.09	6.38	4.2E-09
Ln(occupancy+1)	0.28	0.03	8.49	1.1E-13
Ventilation Type	0.46	0.15	2.99	3.4E-03
Filter Upgrade	-0.24	0.14	-1.73	8.6E-02
PAC Added	-0.72	0.13	-5.31	5.8E-07
Random Effects Variance	Variance	Standard Deviation		
Intercept	0.10	0.32		
Residuals	0.16	0.40		

### 3.7 Energy consumption and cost analysis

The energy and cost analysis results are in Supplementary Table S8. At mechanically ventilated standard schools, enhanced filtration increased energy consumption by ∼1,000 kWh per classroom per year, and the additional overall cost was $9.6 per year per student. At naturally ventilated schools, new PACs required 280 kWh per year per classroom and $7.9 per year per student. At childcare houses, the intervention increased energy consumption by 670 kWh per year per facility, and an additional $10.7 per year per student. These costs are considered low in comparison to school budgets or the costs of absenteeism due to asthma or other PM-related health effects (50).

### 3.8 Teachers’ survey results

Teachers’ and staff responses to selected survey questions are summarized in Table 6. Due to the small sample size, a qualitative evaluation is conducted. Most or nearly all respondents were not dissatisfied with the room cleanness (98%), thermal comfort (80% in cold and warm weather), indoor air quality (84%), and overall IAQ (87%). A sizable fraction (43%–54%) of the teachers and staff reported respiratory or cognitive symptoms that might be related to IAQ issues. Over half of respondents (54%) reported cognitive symptoms, which could be associated with high CO_2_ exposure (87, 88). Ergonomic symptoms were included in the survey as a control measure, as they are believed to have no correlation with IAQ. As expected, only 26% reported ergonomic problems and the percentage was relatively unchanged before and after the intervention. Most respondents (63%) indicated that IAQ had no effect on the students’ learning, suggesting only limited awareness of IAQ significance.

**Table 6:** Teacher’ and staff responses to selected questions. Based on 100 surveys.

	Number of Respondents		Percent of Respondents		Overall	
Response to selected questions	Baseline	Follow-up	Baseline	Follow-up	Number	Percent
*Room Cleanness*						
Not at all clean	0	1	0.0%	2.3%	1	1.0%
Somewhat clean	35	20	61.4%	46.5%	55	55.0%
Very clean	21	22	36.8%	51.2%	43	43.0%
*Are you dissatisfied with*						
Thermal comfort in warmer months	11	7	19.3%	16.3%	18	18.0%
Thermal comfort in cooler months	14	7	24.6%	16.3%	21	21.0%
Overall Air Quality	13	3	22.8%	7.0%	16	16.0%
*Does IAQ interfere/enhance Learning*						
Interferes a lot/somewhat	15	11	26.3%	25.6%	26	26.0%
Enhance a lot/somewhat	6	3	10.5%	7.0%	9	9.0%
No effect	34	29	59.6%	67.4%	63	63.0%
*Overall IAQ*						
Dissatisfied/Very dissatisfied	6	2	10.5%	4.7%	8	8.0%
Neutral	22	14	38.6%	32.6%	36	36.0%
Satisfied/Very satisfied	26	25	45.6%	58.1%	51	51.0%
*Symptoms*						
Respiratory	28	15	49.1%	34.9%	43	43.0%
Cognitive	32	22	56.1%	51.2%	54	54.0%
Ergonomic/pain	16	10	28.1%	23.3%	26	26.0%

## 4 Discussion

### 4.1 IAQ overview

Pollutant levels at the 12 facilities reflect effects of indoor pollutant sources, ventilation (air change rates), filtration, and outdoor pollutant levels. Ventilation rates were generally low (ACRs from 0.9 to 1.4 h^−1^) and well below the recommended minimum of 3 h^−1^ (29). Where occupancy rates were high, e.g., in the standard schools, CO_2_ levels exceeded 1,000 ppm, the ASHRAE guidance level (29). The three facilities with low CO_2_ levels (F5, F8, F10) also had low ACRs (0.9–1.5 h^−1^) but low occupancy. At the facility-level, CO_2_ concentrations averaged from 645 to 1893 ppm, consistent with other studies (28, 51).

Pollutant levels varied considerably across the spaces, reflecting day-to-day, site-to-site, and room-to-room variation. PM_2.5_ mostly fell below the ambient air quality standards (9 μg/m^3^ for annual average and 35 μg/m^3^ for 24-hour average) (89). At baseline, standard schools had higher PM_2.5_ (6.3 ± 3.7 μg/m^3^) and PM_10_ (26.9 ± 13.2 μg/m^3^) compared to the other three facility categories (2.2–3.9 μg/m^3^ for PM_2.5_ and 5.7–7.4 μg/m^3^ for PM_10_). In contrast, at baseline, BC concentrations were lower in standard schools (0.78 ± 0.48 μg/m^3^) than the other three categories (1.04–1.17 μg/m^3^). BC/PM_2.5_ ratios were computed (in the same manner as the PM_2.5_ I/O ratios) as a diagnostic of traffic-related pollution (Supplementary Table S7). This ratio averaged 0.12 in standard schools but much higher, 0.32–0.59, at the other facilities (at baseline for occupied periods). This difference reflects the variations in ambient pollutant levels, since outdoor BC/PM_2.5_ ratios were also lower at standard schools (0.10) and higher at other types of facilities (0.27–0.55, Supplementary Table S6). The nursery had the highest indoor and outdoor BC/PM_2.5_ ratios (0.39–0.55 at baseline), reflecting significant traffic emissions near this facility, which is located on a major arterial road with extensive truck traffic. No cooking occurs at this facility.

PM_2.5_ concentrations measured at the standard schools are comparable with studies conducted elsewhere in the U.S. For example, PM_2.5_ averaged 6.4 ± 0.6 μg/m^3^ in 18 classrooms across 3 urban elementary schools in the northeastern U.S (52); 5.2 ± 2.3 μg/m^3^ in 139 classrooms in 32 schools in another northeast U.S. study (90); and 3–5 μg/m^3^ in four classrooms in a California elementary school (51). PM_2.5_ concentrations in the other three categories are similar to the concentrations measured in British nurseries (0.3–2.3 μg/m^3^) (40). Much higher PM_2.5_ concentrations have been reported in nurseries and elementary schools outside the U.S (10, 28, 91). Measured BC levels were also similar to U.S. studies, which have shown levels from 0.3 to 0.6 μg/m^3^ (52, 90, 92). BC concentrations in the other three facility categories in Detroit were 30% higher than the standard school concentrations, closer to levels measured in Spain (1.3 ± 0.56 μg/m^3^) (91) and Portugal (1.2 μg/m^3^) (93), but still much lower than levels seen in classrooms in polluted regions like northwest China (3.27 ± 1.86 μg/m^3^) (10). A South Korean study reported 0.73 μg/m^3^ of BC in five classrooms across four schools during non-rush hours, similar to the levels in Detroit standard schools; the BC levels increased to 1.53 μg/m^3^ during rush hours (94), illustrating the significance of traffic emissions on indoor BC concentrations.

### 4.2 I/O ratio comparisons

Indoor/outdoor ratios help account for changing outdoor pollutant levels when evaluating the effectiveness of filter interventions and the performance of the single-zone mass balance model. This approach worked well for most standard schools and the nursery, but results were variable and inconsistent for the portable buildings, facilities containing very large rooms, and childcare houses. I/O ratios for PM_2.5_ near one, found at the standard schools, nursery and community centers, suggest a lack of indoor PM_2.5_ sources but also a lack of air cleaning, a poor situation that renders the facility vulnerable to the penetration of outdoor pollution (the outdoor pollution penetration and infiltration can rapidly increase indoor PM levels if air cleaning is absent). High I/O ratios, such as 1.76 ± 2.49 found in the childcare house facilities, indicate potentially significant indoor sources of PM_2.5_, most likely from cooking and re-entrained dust (85, 95, 96).

The filter intervention resulted in significant I/O reductions at three of the four standard school facilities and the nursery; these buildings also showed good agreement between the single-zone model and measurements. Many factors can affect the performance of the intervention and the agreement with models. Key factors governing pollutants at these facilities were captured in the models. The lack of agreement at the other facilities, which included a portable school building (F3), multiuse community centers, and repurposed houses, likely arise from multiple factors, and each building is a special case as discussed below.

Issues with portable buildings used as classrooms have been reported previously, including high CO_2_ concentrations and low ventilation rates (97). Our tests showed that ACRs at the portable building, F3, were comparable to those in the naturally ventilated schools. Portables are typically well sealed spaces with intermittently used wall-mounted HVAC system for each room. Our intervention did not affect the measured PM_2.5_ I/O ratios, possibly because the HVAC system was infrequently operated and windows were opened given the mild outdoor weather in May. In the model, increasing the proportion of untreated outdoor air in the total ventilation supply air (p_n_) from 50 to 71% improved model-measurement agreement significantly (slope = 1.00, R^2^ = 0.79, Supplementary Figure S5A). This demonstrates the need for building-specific parameters, including on-times (duty cycle or schedule) of mechanical systems and window opening times.

Monitoring at one of the community centers, F12, illustrates concerns with smoke events and the associated behaviors that change building operation. While the intervention reduced PM_2.5_ I/O ratios and model results were highly correlated with measurements (Figure 3H), the slope (1.71) was large, possibly due to overestimating p_n_ in this naturally ventilated facility (assumed to be 100%). The baseline and follow-up measurements at this facility were conducted during wildfire smoke events (84) when occupants were instructed to keep the windows closed to reduce entry of smoke. Additionally, ACRs estimated using CO_2_ decay for this large building may represent air exchange between occupied and unoccupied spaces, rather than between occupied spaces and outdoor air. This is supported by the improved model-measurement agreement after reducing p_n_ from 100 to 58% (slope = 0.99, R^2^ = 0.98, Supplementary Figure S5B). At the other two community centers, model assumptions and measurement errors, rather than parameter errors in p_n_, may explain the poor model-measurement agreement (F6 and F7, Figure 3C). These are large and intermittently occupied facilities that contain (temporary) classrooms, multimedia teaching rooms, conference rooms, and large multipurpose open spaces. The larger spaces were likely not well mixed, and IAQ measurements at a single location in a large room may not be representative.

The interventions at the four childcare houses yielded mixed results, and some even had elevated I/O ratios for PM_2.5_ and PM_10_. All of these facilities have kitchens and cooking activities during school hours, which can considerably increase indoor PM_2.5_ levels (85, 95, 98), increasing I/O ratios and potentially masking any improvements due to enhanced filtration. Standard residential kitchens in Detroit do not provide effective localized exhaust that would isolate cooking emissions from other spaces in the building. In addition, re-entrained dust appears to be a significant indoor PM source, demonstrated by time trends and elevated I/O ratios for PM_10_, which reached a value of nearly 4 at F8 and F11. At childcare houses, floor cleaning may be less frequent and efficient than at the other facilities, e.g., schools typically mop and vacuum daily. Moreover, these repurposed older homes were “leaky”, e.g., we saw poorly sealing windows that would promote the penetration of outdoor air without filtration. These factors should be incorporated into models by introducing parameters for indoor sources and multiple zones (37). To address exposure concerns, however, these facilities require both design and operational changes.

### 4.3 Model comparisons

The single-zone box and LMM models shared several input variables, and both demonstrated the effectiveness of enhanced filtration interventions, highlighted the significant contributions of outdoor pollution and indoor PM generation, and revealed the role of key building characteristics. The empirical LMM model shows how several predictors explain indoor PM_2.5_ concentrations while accounting for clustering within rooms and schools. In contrast, the mechanistic mass-balance box model predicts I/O ratios and indoor PM_2.5_ concentrations by simulating physical processes, e.g., outdoor particle penetration, indoor-outdoor air change, and PM deposition and filtration. Unlike the LMM model, the box model did not account for meteorological conditions, occupancy or the clustering of rooms within a facility, however, it did account for the ACR and PAC usage. The major difference between model results was that ACR was a key variable in the single-zone model but was not significant in the LMM model, probably because the ACR was correlated with other variables in the LMM model, e.g., temperature difference, ventilation type, and outdoor PM_2.5_ levels. In addition, the room/school random effects may have captured some ACR-related differences, as rooms within the same facility generally saw similar ACR values. Overall, the LMM model can reveal overlooked and confounding factors that impact IAQ, while the single-zone model enables prediction of IAQ during polluted days and provide a means to evaluate ventilation and filtration strategies.

### 4.4 The importance of teacher/staff behaviors and perspectives

The importance of teacher and staff behaviors and perspectives should be addressed to effectively enhance and maintain IAQ. In the aftermath of Covid-19 pandemic, most child-serving facilities had attempted to improve IAQ using PACs or DIY filtration systems, although few evaluated CADRs and implemented use patterns and maintenance procedures to ensure effectiveness (99). We found very few facilities that had changed PAC filters that were installed 3 to 5 years earlier, well past their lifetime. Infrequent or intermittent operation of the PACs installed or upgraded in this study were common. In most mechanically ventilated buildings, a contractor regularly replaces filters and provides rudimentary HVAC system maintenance. However, filter ratings are generally low (MERV 7 to 11), and we commonly observe improper filter installations, e.g., torn, reversed and missing filters. Additionally, package HVAC and residential style units operate in the “auto” fan setting, meaning that mechanical ventilation – and filtration – occur only when there is a need for heating/cooling.

To improve IAQ, mechanical filtration was upgraded to MERV 13 and supplemented using PACs, but this is only effective if HVAC systems and PACs are operated throughout the day (and potentially earlier) at a sufficient fan speed. The effectiveness of IAQ filter strategies can be impaired by the actions and indifference of teachers and staff. Although teachers were instructed to use PACs throughout the school day, PACs usage was variable (50%–94%) and PACs were operated at the desired high fan speed from 52%–93% of the time. Teachers expressed noise and energy consumption concerns with the PACs and often forget to turn them back on in the morning. While the survey showed that most respondents were satisfied or neutral with IAQ, our assessment identified several IAQ problems. Moreover, over half of respondents were unaware of the effects of IAQ on the students’ learning. These views can diminish motivation for IAQ management, including building and HVAC maintenance and PAC usage. Another concern is the long-term sustainability of the interventions and the associated energy and economic burdens. We estimated annualized costs of about $10 per student per year, somewhat higher than our previous estimate but still lower than the benefits of avoided asthma exacerbations (50). While schools were provided with PACs and HVAC filter replacements for a year, long term follow-up is needed to evaluate maintenance practices and overall sustainability. Similar concerns were raised in a German study that found behavior could confound the effectiveness of PACs in daycares (100), and in another Detroit study of asthma-affected households where PAC use decreased rapidly after initial installation (101).

### 4.5 Facility comparisons and recommendations

The study highlights the diversity of IAQ conditions among different child-serving facilities and the need for tailored improvement strategies. Based on observed IAQ improvements and agreement with the single-zone model, the 12 facilities were divided into three groups for discussion and recommendations. The first group (F1, F2, F4, F5, F12) showed IAQ improvements post-intervention and strong agreement between the measured and single-zone model predicted PM_2.5_ I/O ratio. These facilities had routine HVAC system maintenance, hard flooring (wood or tile) that was cleaned daily, relatively consistent occupancy and school schedules, trained and collaborative teachers and staff, and no indoor cooking. These factors tended to reduce the variability of IAQ levels and minimize indoor PM sources (not quantified in this study). At these facilities, we recommend continuing the daily cleaning, replacing HVAC filters every three months, and operating the HVAC fans and PACs at an acceptable high speed throughout the day (and potentially 30 min to an hour before school starts).

The second group included the childcare houses (F8 – F11) where IAQ measurements were variable and did not agree with model predictions. These facilities are relatively small, the buildings' envelopes are leaky, and the ventilation systems are poorly maintained. We found that the PACs were largely unused, probably exacerbated by a high teacher turnover rate and inconsistent occupancy and class schedules. Additional common factors included cooking without effective exhaust ventilation, rooms with carpets, and less frequent cleaning (once or twice a week). These practices increased indoor PM generation and dust resuspension. At these facilities, we suggest better sealing of windows and doors, replacing furnace filters every three months, daily cleaning of hard and carpeted floors, and increasing use of PACs, particularly during cooking or indoor play activities.

The third group (F3, F6, F7) were facilities that were distinct and had a number of limitations to the dataset that preclude meaningful generalizations. F3 was the only portable facility, and our results had large uncertainties largely due to possible window openings and infrequent HVAC operations. We suggest that the HVAC system operates throughout the school days, noisy fans (observed for one unit) be repaired, and that daily cleaning be maintained (80). The large, multipurpose F6 and F7 facilities did not receive enhanced filtration. We noticed poor mixing in the larger rooms, and potentially significant PM emissions from the robotics workshop and 3D printing at F6. We suggest that these facilities use exhaust ventilation and PACs or other controls near indoor sources, isolate space uses, and use additional fans to enhance mixing in the common rooms (without the workshop).

Across the facilities, we also note the importance of training and education regarding energy use, economic costs, and IAQ. This can inform and benefit beliefs and perspectives of teachers and staff, which can influence behaviors related to promoting ventilation and operating PACs. This may be challenging at the childcare houses due to the high staff turnover.

### 4.6 Limitations

The single-zone model used in this research does not reflect the complexity of many indoor environments. Important uncertainties and errors include the omission of indoor emission sources, e.g., from cooking and dust resuspension, which can elevate indoor PM_2.5_ concentrations, suggest the penetration of outdoor pollutants, and mask the evaluation of enhanced filtration. This model also assumed steady-state conditions, single particle size and constant mixing factors. These assumptions may not fully capture the temporal and spatial variability of PM_2.5_ concentrations, particularly in the childcare houses where occupancy, indoor activities and ventilation patterns may vary substantially, and in the large rooms of community centers due to incomplete mixing and stagnation. To provide a more comprehensive assessment of factors affecting filtration, future studies might incorporate activity logs, sensors to monitor window/door openings, multiple sites to characterize IAQ in large rooms, and building and room-specific characteristics, e.g., leakage and HVAC operation schedules. Wildfire smoke events were not systematically evaluated. These events were not identified as a priority during the study design. Only one severe event occurred in the study, specifically, the follow-up measurements at F12 coincided with an unexpected and severe smoke event, which significantly elevated indoor and outdoor PM_2.5_ levels, as shown in Table 2 and S6. Still, the I/O ratios remained strongly correlated with predictions from the single-zone model. Future applications in regions that experience frequent wildfire smoke events might use similar methods to estimate IAQ and PAC performance. In such cases where outdoor pollutant sources are so dominant, model performance may improve since the omission of even strong (but uncertain) indoor sources becomes less critical. At the same time, wildfire smoke may differ in particle size distribution, dynamics (temporal patterns), and building envelope penetration points from typical urban pollutants, e.g., traffic-related pollutants that enter rooms near major roads and parking areas during morning and afternoon periods. These characteristics, especially smoke's more monodispersed size distribution (102, 103), may affect the performance of filters. Thus, additional research is needed to evaluate the effectiveness of filtration at schools undergoing frequent wildfire smoke events.

## 5 Conclusions

Baseline and follow-up IAQ measurements, and teacher and staff surveys were conducted for a filtration intervention in 12 diverse child-serving facilities. Overall, we found acceptable temperatures and humidity, low ventilation rates, high CO_2_ concentrations, and PM_2.5_ and black carbon concentrations that tracked or exceeded outdoor levels. The intervention attained moderate reductions of PM_2.5_ and PM_10_ concentrations in a subset of the facilities, reductions that were expected and consistent with predictions from a single-zone mass balance mode and a LMM model. However, buildings that included very large rooms, portable classrooms, and repurposed residences did not attain significant IAQ improvements. While most teachers and staff were satisfied with indoor cleanness, thermal comfort and overall IAQ, they were unaware of IAQ issues and their potential impact on classroom learning. The study highlights the diversity of conditions found in buildings used as child-serving facilities, particularly those buildings that have been reconfigured to serve this function without explicitly addressing IAQ. We also highlight the needs for filter and HVAC configurations and operational modes that meet minimum clean air delivery rates throughout the building, and the role of teacher and staff education regarding IAQ that can help promote the long-term sustainability of filter interventions. Ultimately, both building-specific management and maintenance, as well as appropriate training and education for teachers and staff, are needed to achieve and maintain meaningful IAQ improvements.

## Data Availability

Relevant data acquired in the study are included in the article/Supplementary Material, further inquiries can be directed to the corresponding author.
