# Supplementary material for: Effectiveness of enhanced filtration interventions for improving indoor air quality in child-serving facilities
Source: Front Pediatr. 2026 Aug 4;14:1875263. doi: 10.3389/fped.2026.1875263 (PMC13481836; doi:10.3389/fped.2026.1875263)
Supplement: Supplementary file 1 [file Supplementaryfile1.docx]

**Effectiveness of Enhanced Filtration Interventions for Improving Indoor Air Quality in Child-Serving Facilities**

Submitted to *Frontiers in Pediatrics*

Special issue: Indoor Environmental Quality in Day-Care Centers, Kindergartens, and Primary Schools

https://www.frontiersin.org/research-topics/67300/indoor-environmental-quality-in-day-care-centers-kindergartens-and-primary-schools

July 9, 2026

Tian Xia^1^, Julia Raneses^1^, Morgan Locke^2^, Raquel Garcia^3^, Alison Walding^1^, Ricardo F. DeMajo^2^, Amy J. Schulz^2^, Stuart A Batterman^1^

^1^Environmental Health Sciences, School of Public Health, University of Michigan, Ann Arbor, MI, US

^2^Health Behavior and Health Equity, School of Public Health, University of Michigan, Ann Arbor, MI, US

^3^Southwest Detroit Environmental Vision, Executive Director, Detroit, MI, US

# 1 Interventions

Table 1 in the main paper describes baseline and intervention filtration at the 12 facilities. The intervention goal was to attain an effective clean air change rate (ACR plus clean air change per hour due to PAC usage) of between 3 and 6 hr^-1^ in each room, following ASHRAE guidance (1). As discussed in the paper, this goal was difficult to achieve due to infrequent PAC usage and issues with facility, PAC and HVAC system maintenance.

In facilities without central ventilation (F1, F4, F5, F11 and F12), 1 – 3 PACs were installed or serviced (filter replacement and usage monitoring) in each room after the baseline monitoring. Teachers and staff were told to run the PACs at the highest setting they could tolerate without noise issues during occupied hours, while running the PAC overnight or during unoccupied hours (weekends and holidays) were optional. A smart plug (Eve Energy, Munich, Germany) monitored the power usage of each air purifier for every 10 minutes. In each of the eight classrooms in F1, under the wall opposite the existing PAC (MA-40, Medify, Boca Raton, FL, USA), one Whispure PAC (Whispure, Honeywell, Charlotte, NC, USA) was deployed. Additionally, three new Medify air purifiers were installed in the other three rooms so that all rooms in F1 have two PACs. The Whispure PAC have 4 speeds (low, medium, high, turbo) and the corresponding CADRs were measured to be 221, 281, 320 and 404 ft^3^/min (375, 477, 544 and 686 m^3^/hr) (2); the Medify units have 3 speed settings (low, medium, high) with the corresponding CADRs of 144, 181, and 239 ft^3^/min (245, 308, and 406 m^3^/hr), respectively (3). Two Whispure purifiers were installed on opposite sides (usually front and back) in each of the 10 regular classrooms in F4. F5 already had one PAC (MA-50, Medify, Boca Raton, FL, USA) installed in each of the five childcare rooms. The PAC has four speeds at 24, 105, 165, and 340 ft^3^/min (41, 178, 280, and 578 m^3^/hr) CADR, respectively, as measured by the study team. None of them, however, had the HEPA filter replaced since first installation (> 2 years), and the filters were loaded with dust. The intervention involved replacing the filters for all units. F11 and F12 had one PAC (EE-5068, Crane, Itasca, IL, USA) installed in selected rooms, while the PACs were seldom used and the HEPA filters were never changed. The PAC has three speeds at 11, 21, and 39 ft^3^/min (19, 36, and 66 m^3^/hr) CADR, respectively, as measured by the study team. To enhance filtration, the number of PACs in the monitored rooms were increased to 2 – 3 (according to the room size) and all filters were replaced. The PACs and replacement filters were donated to each facility at the end of the intervention period.

In the mechanically ventilated facilities (F2, F3, F8, F9, F10), the intervention upgraded HVAC filters to MERV 13. Two facilities (F2, F3), already had a Medify MA-40 PAC installed at the front of each classroom before the project started. At these buildings, we replaced filters and monitored their usage. At two other facilities (F6 and F7), interventions and follow-up measurements were not conducted given infrequent use of the child-serving rooms and budget constraints. PAC purchases or filter upgrades in these large spaces (37,509 cubic feet (1062 m^3^) at F6 and 14,057 cubic feet (398 m^3^) at F7) would have exceeded our budget.

# 2 Single zone IEQ model

The fundamental equation for the single zone IEQ model is:

$\frac{\mathrm{VdC}}{\mathrm{dt}}=Q C_{0}-Q C-k_{PAC} {\eta CQ}_{c}- {k_{D}\Sigma v}_{d,i} A_{i} C$	(1)

where V = room volume, C = PM_2.5_ concentration, t = time, Q = ventilation air flow rate, C_0_ = outdoor PM_2.5_ concentration, k_PAC_ = mixing factor for PAC operation, k_D_ = mixing factor for PM_2.5_ deposition, η = PAC purifier/HVAC filter PM_2.5_ removal efficiency (assumed to be 99.97% for all HEPA filters (4)), Q_c_ = air purifier CADR, v_d,i_ = deposition velocity to surface i, and A_i_ = deposition area of surface i. Measured room dimensions and volumes were used. The mixing factor indicates the fraction of PM_2.5_ completely mixed with room air (usually 0.33 – 1.00) (58) during filtration or deposition.

Eq. 1 can be transformed to:

$\frac{\mathrm{dC}}{\mathrm{dt}}={{[p}_{n}+\left( 1-p_{n} \right) \eta] r}_{1} C_{0}-\left( r_{1}+k_{PAC} {\eta r}_{2}+k_{D} D \right) C$	(2)

where r_1_ = room ACR, r_2_ = clean air delivery per hour (Q_c_/V) of the air purifiers (for rooms with PACs, r_2_ = 0 at baseline and was determined by the smart plug measurements at follow-up), p_n_ = percent of untreated outdoor air in the total air change, and D = overall deposition rate, defined by:

$D= \frac{v_{d,f}A_{f} + v_{d,w} A_{w} + v_{d,c} A_{c}}{V}$	(3)

where v_d_ = particle deposition velocity, A = surface area, and subscripts f, w and c represent the room floor, walls, and ceiling, respectively. All particles were assumed to be 2.5 μm in diameter and the friction velocity was assumed to be 0.3 m/s (5). Lai and Nazaroff (6) suggest a friction velocity of at least 0.1 m/s, and we selected 0.3 m/s (7). The assumed large diameter and high friction velocity represents a relatively high deposition scenario for PM_2.5_.

At steady state, dC/dt = 0, and the PM_2.5_ I/O ratio can be estimated using the following equation:

I/O ratio = $\frac{C}{C_{0}}= \frac{{[p}_{n}+\left( 1-p_{n} \right) (1-\eta)] r_{1}}{r_{1}+k_{PAC} {\eta r}_{2}+k_{D} D}$	(4)

Linear regressions between predicted and estimated PM_2.5_ I/O ratios suggested the performance of the single zone model and illustrate the impact of room variables, ACR, and PAC usage (if applicable) on the IEQ improvement effectiveness. To improve model performance and reduce the assumption uncertainties, a sensitivity analysis was conducted. The results indicated that the assumptions that half of the ventilation is from outdoor air for mechanically ventilated facilities (p_n_ = 50%), and k_PAC_ and k_D_ = 0.75 worked best (the largest r^2^ in linear correlations) in this study (the sensitivity analysis is detailed below).

# 3 Sensitivity Analysis

The sensitivity study used PM_2.5_ measurements at the standard school facilities (F1 – F4, after excluding one outlier as discussed in Section 3.5 in the main paper) and was aimed at improving model and measurement agreements.

## 3.1 Data Screening Criteria

The first sensitivity study examined different data screening criteria (lower and upper limits). Six tests were conducted with the parameters listed below. The goal of this test is to examine if the lower limit should be increased, or if setting up an upper limit is necessary. Note that in the listed six tests both outdoor and indoor measurements used indoor percentiles as the limit values. The results showed that the 0.5^th^ percentile as a lower limit is acceptable, but the upper limit should be increased (Test 4 vs. Test 1). After further examination, we found that using the 0.5^th^ and 99.5^th^ percentiles is a suitable approach, while we need to use the indoor percentiles for indoor measurements and outdoor percentiles for outdoor measurements. After adopting this approach, the final results in Fig. S1G yielded the best model-measurement agreements.

Table S1. List of the parameters tested in the data screening criteria sensitivity analysis.

D) Test 4

C) Test 3

B) Test 2

A) Test 1

F) Test 6

E) Test 5

G) Final

Figure S1. Sensitivity test on the data screening criteria.

## 3.2 Outdoor Concentration

Outdoor PM_2.5_ levels were estimated in four methods. The primary method (Test 1) applied our local measurements wherever they are available and used the PM_2.5_ concentrations averaged over five southwest Detroit government stations (labeled as SW) for five child-serving facilities that did not have local outdoor measurements. Test 2 used the hourly SW government station measurements for all facilities. Five facilities, F5, F7, F8, F9, and F11 are not located in southwest Detroit. F5 and F7 are in east Detroit and the PM_2.5_ concentrations averaged over two east Detroit government stations (labeled as E) were used for them in Test 3 and 4. F8, F9 and F11 have no nearby monitoring stations. Test 3 used the measurements from one “background” station near Detroit, AP, to represent the low outdoor concentration scenario, and Test 4 used the measurements from one near-road station, EH, to represent the high outdoor concentration scenario. The results showed that Test 2 – 4 yielded almost identical correlation plots, and the model-measurement agreements were slightly better than Test 1 (slope: 0.97 vs. 0.92; R^2^: 0.83 vs. 0.79). Since Test 1 still yielded good agreements and we wanted to use our own measurements in most of the analysis, we used the Test 1 method to estimate outdoor PM_2.5_ concentrations.

Table S2. List of the parameters tested in the outdoor PM_2.5_ concentration sensitivity analysis.

B) Test 2

A) Test 1

D) Test 4

C) Test 3

Figure S2. Sensitivity test on the outdoor PM_2.5_ concentration estimation methods

## 3.3 Percentage of untreated outdoor air in total ventilation

We tested four assumptions for the percentage of untreated outdoor air in the total air change (p_n_), as listed in the table below. Tests 1, 2 and 4 showed similar results and we chose to use our original assumption, 100% and 50% of outdoor untreated air in the total air change (p_n_) for naturally and mechanically ventilated facilities, respectively.

Table S3. List of the parameters tested in the percentage of outdoor air ventilation (p_n_) sensitivity analysis.

B) Test 2

A) Test 1

D) Test 4

C) Test 3

Figure S3. Sensitivity test on the percentage of outdoor air ventilation (p_n_).

## 3.4 Mixing Factors

We tested three assumptions for the mixing factor, as listed in the table below. The medium mixing scenario, with a mixing factor of 0.75, yielded the best model-measurement agreement and we used that value for our final model.

Table S4. List of the parameters tested in the mixing factor sensitivity analysis.

| Mixing Factor | |
| --- | --- |
| High | 1.00 |
| Med | 0.75 |
| Low | 0.50 |
|  |  |

C) Low

B) Med

A) High

Figure S4. Sensitivity test on the mixing factor.

## 3.5 Improved model-measurement agreement by adjusting p_n_ for F3 and F12

Figure S5. Improved model-measurement agreement at A) F3 by increasing p_n_ in the model from 50% to 71%; and B) F12 by reducing p_n_ from 100% to 58%.

# 4 Additional tables and figures

Table S5. Average room area, height, and PAC location (if applicable) for each participating child-serving facility

Table S6. Summary of outdoor PM_2.5_, PM_10_, BC concentrations and BC/PM_2.5_ ratios for four facility categories during occupied and unoccupied periods during the baseline and follow-up sampling. Data marked red are outdoor measurements collected by government monitoring sites during a wildfire smoke event in 2025.

Table S7. Indoor BC/PM_2.5_ ratios for four facility categories during occupied and unoccupied periods during the baseline and follow-up sampling.

Table S8. Estimated energy and economic cost for enhanced filtration interventions. All costs were estimated based on the current prices, assuming no inflation rates.

|  |  | Pre-intervention | Post-intervention |
| --- | --- | --- | --- |
| *HVAC system* | |  |  |
|  | Filters per unit^1^ | 6 | 6 |
|  | Filter price ($)^2^ | 10.0 | 19.0 |
|  | Replacement per school year^3^ | 3 | 3 |
|  | **Annual filter cost ($)** | **180.0** | **342.0** |
|  | Fan power (W)^1^ | 3383 | 3383 |
|  | School days^4^ | 177 | 177 |
|  | Runing time per school day (h)^4^ | 3.3 | 10.0 |
|  | Electricity price ($/kWh)^5^ | 0.2 | 0.2 |
|  | **Annual energy consumption (kWh)** | **1976** | **5988** |
|  | **Total energy cost per year ($)** | **395.2** | **1197.6** |
|  | **Total annual cost ($)** | **575.2** | **1539.6** |
|  | **Cost per year per classroom ($)^6^** | **143.8** | **384.9** |
|  | **Cost per year per student ($)^6^** | **5.8** | **15.4** |
| *PAC (Whirlpool WP500 Whispure HEPA Air Purifier)* | | |  |
|  | Number of units^7^ | 0 | 2 |
|  | Unit price ($)^8^ |  | 275 |
|  | Power (W)^7^ |  | 100 |
|  | School days^4^ |  | 177 |
|  | Runing time per school day (h)^4^ |  | 8.0 |
|  | Electricity price ($/kWh)^5^ |  | 0.2 |
|  | Filter replacement per year^7^ |  | 1 |
|  | Filter price ($)^9^ |  | 43.0 |
|  | **Annual energy consumption (kWh)** |  | **283.2** |
|  | **Annualized capital cost ($)^10^** |  | **55.0** |
|  | **Cost per year per student ($)** | **0.0** | **7.9** |
| *Forced-air system for childcare houses* | |  |  |
|  | Filter price ($)^2^ | 6.7 | 9.7 |
|  | Replacement per year^11^ | 2 | 4 |
|  | **Annual filter cost ($)** | **13.4** | **38.8** |
|  | Fan power (W)^4^ | 500 | 500 |
|  | School days^4^ | 240 | 240 |
|  | Runing time per school day (h)^4^ | 2.4 | 8.0 |
|  | Electricity price ($/kWh)^5^ | 0.2 | 0.2 |
|  | **Annual energy consumption (kWh)** | **288** | **960** |
|  | **Total energy cost per year ($)** | **57.6** | **192.0** |
|  | **Total annual cost ($)** | **71.0** | **230.8** |
|  | **Cost per year per student ($)^12^** | **4.7** | **15.4** |

^1^ We assumed a 12.5-ton Trane rooftop AHU was installed. The AHU information is from <https://www.trane.com/content/dam/Trane/Commercial/global/products-systems/equipment/unitary/rooftop-systems/precedent-3-to-25-tons/RT-PRC107A-EN_12022022.pdf>

^2^ <https://filterbuy.com/air-filters/>

^3^ Assum the filters were replaced every 3 months during the school year (9 months)

^4^ Values estimated according to a previous study (8).

^5^ <https://findenergy.com/mi/wayne-county-electricity/>

^6^ Assume one AHU unit serves 4 classrooms. Each classroom has 25 students.

^7^ Based on our estimations, each regular classroom needs 2 PACs to achieve desired CADR. The PAC power at the highest fan speed is 100W, and the instruction manual suggests one filter replacement per year.

^8^ <https://www.amazon.com/Whirlpool-WP500B-WP500-Whispure-Purifier/dp/B076TF2NDX>

^9^ <https://www.amazon.com/Pureburg-Replacement-Pre-Filters-Whirlpool-AP51030KB/dp/B07PJQP5H1>

^10^ Assume the lifetime of the PAC is ten years, so the annualized cost per unit is 1/10 of the current unit price.

^11^ Assume the filters were replaced twice per year before intervention and every three months as we suggested after intervention.

^12^ Based on our observations, assume each childcare house has 15 students.

Table S9. LMM results of the primary model with ln(PM_2.5,in_) as the dependent variable and 12 fixed-effect variables.

Table S10. LMM results of the final model with ln(PM_2.5__I/O ratio) as the dependent variable and eight fixed-effect variables (indoor-outdoor temperature difference, outdoor RH, ln(occupancy +1), ventilation type, filter upgrade, and PAC added).

Table S11. Multi-linear regression model evaluating impact of methodological conditions, i.e., temperature, RH, wind speed, sin(wind direction) and cos(wind direction), on the measured outdoor PM_2.5_ concentrations. Only the outdoor RH showed significant impact on the ambient PM_2.5_ level.

7)

6)

5)

4)

3)

2)

1)

Figure S6. Photo of the instruments inside the sampler case (left) and sampler setup (right), including 1) sound pressure level meter (SPL); 2) CO_2_ sensor; 3) optical particle counter; 4) MA200 aethalometer for BC measurements; 5) T/RH sensor; 6) DSTech PM and BC sensor; and 7) microphone.

# 5 Survey Questionnaire

**Default Question Block**

WELCOME!

Welcome to the Community Action to Promote Healthy Environments (CAPHE) Teacher Survey. CAPHE is a partnership between community-based organizations (48217, Detroit Hispanic Development Corporation, Detroiters Working for Environmental Justice, Ecology Center, Greendoor Initiative,Southwest Detroit Community Benefits Coalition, Southwest Detroit Environmental Vision), government health and environmental organizations (Detroit Health Department, Michigan Environment, Great Lakes and Energy), and researchers based in academic institutions (University of Detroit Mercy, University of Michigan Dearborn, University of Michigan School of Public Health). Our focus is on reducing air pollution and its adverse health effects in Detroit and surrounding [communities.(https://caphedetroit.sph.umich.edu/).](https://caphedetroit.sph.umich.edu/)) Our Schools Indoor Air Project (ScIP) is placing indoor air filters in selected child-serving organizations in Detroit and surrounding areas, and measuring reductions in indoor air pollution and selected educational outcomes (e.g. reductions in school absenteeism associated with asthma).

Your responses on this brief survey will provide important information to inform future actions to improve air quality in child serving organizations. We greatly value your input and thank you for your participation.

Consent to participate in a Research Study – School Indoor Air Quality: Teacher Survey. eResearch ID #HUM00202727

On behalf of the CAPHE Partnership, we invite you to participate in a research study that examines environmental conditions in indoor spaces where children spend time and are

learning. This form contains information that will help you decide whether to join the study. The purpose of this research is to evaluate the impacts of placing indoor air filters in spaces where children spend time (e.g. schools, child-care centers) on air quality and selected educational indicators (e.g., school absenteeism), and to inform efforts to improve indoor environmental quality and create comfortable learning environments for Detroit area students. This study is funded by the U.S. National Institute for Environmental Health Sciences. We are asking you to participate because you work with young people in a classroom or indoor space. We are asking you to participate because you work with young people in a classroom or other indoor space . If you agree to participate in this research study, you will be asked to complete 2 short surveys – one before indoor air filters are installed, and once again after they are installed about your satisfaction with the environmental conditions of your indoor space This survey should take less than 10 minutes to complete. You will not be paid for participating in this study. While you may not receive any direct benefit for participating, we hope that this study will contribute to improved indoor environmental quality and educational environments in your building and other Detroit-area organizations that serve children. Researchers will not be able to link your survey responses to you, but they will know that you participated in the research because you will be asked to log in. We will be asking you to complete the survey two times (before and after the filters are installed in your building or room) and we will include a code number that links your responses at those two points in time, without identifying you. The survey software keeps your identifying information other than the linking code, separate from your answers. We plan to publish the results of this study, but we will not include any information that would identify you, your room, or your organization.

Participating in this study is completely voluntary. You do not have to participate, and you can stop at any time. Even if you decide to participate now, you may change your mind and stop at any time. You may choose to not answer an individual question, or you may skip any section of the survey. Simply click “Next” at the bottom of the survey page to move to the next question. The survey should be taken in a private space. This project has been reviewed by the University of Michigan Health Sciences Behavioral Science Institutional Review Board (IRB). This research holds a Certificate of Confidentiality from the National Institutes of Health. This means that we cannot release or use information, documents, or samples that may identify you in any action or suit except as described below. This protection includes federal, state, or local civil, criminal, administrative, legislative, or other legal proceedings. An example of a situation in which the Certificate would apply would be a court subpoena for research records. There are some important

things that you need to know: ● The Certificate does not stop reporting or information-sharing that you agreed to in this consent document. For example, we may share information from the survey with other researchers. ● The Certificate cannot be used to stop a sponsoring United States federal or state government agency from checking records or evaluating programs. ● We may also release information about you to others when you say it is okay. For example, you may give us permission to release information to insurers, medical providers, or anyone else. ● The Certificate of Confidentiality does not stop you from personally releasing information about your involvement in this research if you wish. More information about Certificates of Confidentiality and the protections they provide is available at [https://grants.nih.gov/policy/humansubjects/coc/what-is.htm.](https://grants.nih.gov/policy/humansubjects/coc/what-is.htm) If you have questions about this research study, you can contact Dr. Stuart Batterman, School of Public Health, University of Michigan, Ann Arbor, MI, 48109-2029, (734-763-2417) [stuartb@umich.edu,](mailto:stuartb@umich.edu) Dr. Amy Schulz, School of Public Health, University of Michigan Ann Arbor, MI, 48109-2021 (734-647-0221), [ajschulz@umich.edu](mailto:ajschulz@umich.edu) or Alison Walding, School of Public Health, University of Michigan, Ann Arbor, MI 48109-2021 (515-240-7904), [walison@umich.edu.](mailto:walison@umich.edu) If you would like a copy of the consent, please feel free to print this page. If you are not able to print, please contact Alison Walding [walison@umich.edu](mailto:walison@umich.edu) to receive an emailed copy. You may also express a question or concern about this study by contacting the Institutional Review Board listed below: University of Michigan Health and Behavioral Sciences, 2800 Plymouth Road, Bldg. 520, Rm. 1169, Ann Arbor, MI 48109-2800, (734) 936-0933, [irbhsbs@umich.edu](mailto:irbhsbs@umich.edu) By clicking on “I agree” you are consenting to participate in this research survey.

I agree	I do not agree

About your room: Please answer the following questions about the room in which you offer activities for children or youth. This may be a classroom, a child-care center, a community based organization that offers after school activities for children or youth.

Name of your organization

Room name/number if applicable

2. Please indicate how often each of the following takes place in the room that you use.

| Never | Rarely | Sometimes | Frequently | Constantly |
| --- | --- | --- | --- | --- |
| a. Art activities  |  |  |  |  |
| b. Science activities  |  |  |  |  |
| c. Industrial/vocational activities  |  |  |  |  |

3. Are food and drink allowed in this room? Yes

 No

4. Please check which, if any, of the following are kept, stored or used in the room that you use. [check all that apply]:
   a. outdoor clothing (raincoats, snow boots, warm jackets)
   b. sportswear (gym clothes, swimsuits, towels)
   c. plants
   d. animals/pets
   e. pet supplies
   f. markers
   g. paint
   h. glue
   i. science materials
   j. cleaning chemicals
   k. food
   l. other (please write in)
   m. None of the above
5. How would you rate the general cleanliness of the room? Not at all clean

 Somewhat clean Very clean

6. Does your room have any of the following? (please check all that apply)
   a. Operable windows (can be opened)
   b. Door to the outside
   c. Air fresheners (candles, scents, deodorizes, plug-in units)
   d. Sink
   e. None of the above
7. Does your room have a stand-alone air purifier? Yes

 No

8. Is the air filter usually turned on? Always

 Most of the time Sometimes

 Rarely Never

Please describe the reasons why the air purifier is not being used regularly.

9. Does your organization explicitly forbid teachers opening or closing windows? Yes

 No

 Don't know

**Block 1**

THERMAL COMFORT

The following questions ask for your views about thermal comfort in the room (not your students' views).

10. Which of the following do you control in the meeting room or classroom? Please check all that apply
    a. Thermostat
    b. Opening windows
    c. Ceiling fan
    d. Adjustable air vents
    e. Adjustable blinds or shades
    f. Door to interior
    g. Room air conditioner
    h. Door to exterior
    i. Permanent heater
    j. Portable heater
    k. Portable fan
    l. Other
    m. None of the above
11. How satisfied are you with the thermal comfort in this room in the warmer months?

 Very dissatisfied Dissatisfied

 Neutral Satisfied

 Very satisfied

12. In the warmer months, do any of the following sources of discomfort occur in this room? Please check all that apply

Temperature

a. Too warm
b. Too cool
c. Temperature varies a lot over the day Humidity
d. Too dry
e. Too humid
f. Thermostat does not respond or cannot be controlled adequately
g. Walls or floor too cold/warm
h. Drafts from windows
i. Heat gain from windows
j. Lack of windows that open
k. Drafts from vents
l. Too little/too much ventilation (air flow)
m. Clothing policy is not flexible
n. None of the above

13. How satisfied are you with the thermal comfort in this room in the cooler months?
    a. Very dissatisfied
    b. Dissatisfied
    c. Neutral
    d. Satisfied
    e. Very satisfied
14. In the cooler months, do any of the following sources of discomfort occur in this room? Please check all that apply

Temperature

a. Too warm
b. Too cool
c. Temperature varies a lot over the day Humidity
d. Too dry
e. Too humid
f. Thermostat does not respond or cannot be controlled adequately
g. Walls or floor too cold/warm
h. Drafts from windows
i. Heat gain from windows
j. Lack of windows that open
k. Drafts from vents
l. Too little/too much ventilation (air flow)
m. Clothing policy is not flexible
n. None of the above

15. Overall does the thermal comfort in this room interfere with or enhance learning? Interferes a lot

Interferes somewhat No effect

Enhances somewhat Enhances a lot

**Block 2**

AIR QUALITY

16. How satisfied are you with the air quality (freshness of air, stuffy/stale air, odors) in this room?

Very dissatisfied Dissatisfied

Neutral Satisfied

Very satisfied

17. Please estimate how often you have experienced any of the following odors in this room.

| Never | Rarely | Sometimes | Frequently | Most of the time | Constantly |
| --- | --- | --- | --- | --- | --- |
| a. perfume/cologne |  |  |  |  |  |
| b. musty odor |  |  |  |  |  |
| c. food |  |  |  |  |  |
| d. carpet odors |  |  |  |  |  |
| e. burnt odor |  |  |  |  |  |
| f. paint varnish |  |  |  |  |  |
| g. tobacco smoke |  |  |  |  |  |
| h. cleaning products |  |  |  |  |  |
| i. chlorine/swimming pool |  |  |  |  |  |
| j. body odor/stale air |  |  |  |  |  |
| k. pesticides |  |  |  |  |  |
| l. sewer gas |  |  |  |  |  |
| m. freshly cut grass |  |  |  |  |  |

Never	Rarely	Sometimes Frequently

Most of the

time	Constantly

n. raw gasoline

o. automobile exhaust

p. diesel exhaust

q. asphalt

r. other unpleasant odors (please describe)

18. Overall, does the air quality interfere with or enhance learning in this room? Interferes a lot

Interferes somewhat No effect

Enhances somewhat Enhances a lot

**Block 3**

NOISE

19. How satisfied are you with the level of noise in this room?

Very dissatisfied Dissatisfied

Neither satisfied nor dissatisfied Satisfied

Very satisfied

20. Which, if any, of the following contribute to your lack of satisfaction with noise levels

in this room? [please check all that apply]

a. Outdoor noise due to traffic, trucks, buses, etc.
b. Noise from indoor air filter
c. Noise from ventilation system or fans
d. Noise from children and classroom activities due to echoes and reflections
e. Noise from nearby classrooms or hallways
f. None of the above

**Block 4**

OVERALL CLASSROOM ENVIRONMENT

21. Overall, how satisfied are you with the environmental quality in this room to encourage learning?

Very dissatisfied Dissatisfied

Neutral Satisfied

Very satisfied

22. Please describe the reasons that you are dissatisfied with the overall environmental quality in this room to encourage learning (if not listed before)

**Block 5**

ABOUT YOU

23. About how many hours do you spend inside this room on a typical day?
    a. 0-2

b. 3-4

c. 5-6

d. 7-8

e. More than 8

24. How many years have you been working in this room?
    a. Less than 1 b. 1-2

c. 3-4

d. 5 or more

25. Please indicate your year of birth

Year (1900-2015)

26. Please indicate your gender
    a. Male
    b. Female
    c. Non-binary
    d. Other
    e. Prefer not to disclose
27. Do you currently smoke? Yes

No

28. Do any other members of your household currently smoke? Yes

No

29. Which, if any, of the following animals do you keep in your home? (please check all that apply)
    a. Dogs
    b. Cats
    c. Birds
    d. Other:
    e. None of the above
30. The following asks about symptoms or discomforts you may experience at your organization. Please exclude symptoms associated with the common cold. Which of the following to you experience while at your organization (please check all that apply):
    a. Headache
    b. Tiredness/fatigue
    c. Coughing
    d. Shortness of breath
    e. Difficulty concentrating
    f. Hand pain and discomfort
    g. Nausea
    h. Irritation of eyes, nose and throat
    i. Sneezing
    j. Blurred vision
    k. Back pain and discomfort
    l. Wrist pain and discomfort
    m. Dizziness
    n. Breathing problems
    o. Wheezing
    p. Sinus congestion
    q. Neck pain and discomfort
    r. Shoulder pain and discomfort
    s. Other (please describe):
    t. None of the above
31. Have you seen a doctor for any of these symptoms? Yes

No

32. On average, when you experience these symptoms, how long have you been at work/in the building? If the time varies depending on the symptom, please just give us your best estimate of the length of time, on average.
    a. 1 hour or less
    b. 2-4 hours
    c. More than 4 hours
33. On average, when do those symptoms go away? If the time varies depending on the symptom, please just give us your best estimate of the length of time, on average.
    a. Overnight
    b. After a week away
    c. Rarely/never
34. Has the pain or discomfort caused by any of these symptoms caused you to take time off from work?

Yes No

35. Do you have any other health conditions that may make these symptoms worse? (please check all that apply)
    a. Allergies
    b. Immune system disorders (e.g., lupus, arthritis)
    c. Chronic cardiovascular or lung disease
    d. Other (please specify)
    e. None of the above
36. Please write any comments or questions about the survey in the space below.

Do you consider yourself to be Hispanic or Latino or of Spanish origin? Yes

No

What race do you consider yourself?

Asian (e.g., Chinese, Vietnamese, Korean Black/African American

Native American/Alaska Nataive

Native Hawaiian or other Pacific Islander (e.g., Samoan, Chamorro) White

Some other race [please print race or area of origin]

This is the end of the survey. Thank you very much for your time in helping us to understand the indoor environment at your organization and its implications for learning and the health of teachers, other staff and students.

[Powered by Qualtrics](http://www.qualtrics.com/)

# 6 References

1.	ASHRAE. Design Guidance for Education Facilities: Prioritization for Advanced Indoor Air Quality [Internet]. Available from: chrome-extension://efaidnbmnnnibpcajpcglclefindmkaj/https://www.ashrae.org/file%20library/technical%20resources/free%20resources/design-guidance-for-education-facilities.pdf

2.	Batterman S, Godwin C, Jia C. Long Duration Tests of Room Air Filters in Cigarette Smokers’ Homes. Environ Sci Technol. 2005 Sep 1;39(18):7260–8. doi:10.1021/es048951q

3.	MA-40 Air Purifier for Home, School & Offices [Internet]. [cited 2024 Mar 21]. Available from: https://medifyair.com/products/medify-ma-40

4.	Hoang A, Heming A. Preparation in the pandemic: How schools implemented air quality measures to protect occupants from COVID-19. World Health Organ Geneva Switz. 2021.

5.	Ng L, Poppendieck D, Polidoro B, Dols WS, Emmerich S, Persily AK. Single-Zone Simulations Using FaTIMA for Reducing Aerosol Exposure in Educational Spaces. NIST [Internet]. 2021 Apr 8 [cited 2024 Feb 14]. Available from: https://www.nist.gov/publications/single-zone-simulations-using-fatima-reducing-aerosol-exposure-educational-spaces

6.	Lai ACK, Nazaroff WW. MODELING INDOOR PARTICLE DEPOSITION FROM TURBULENT FLOW ONTO SMOOTH SURFACES. J Aerosol Sci. 2000 Apr 1;31(4):463–76. doi:10.1016/S0021-8502(99)00536-4

7.	Xia T, Raneses J, Schmiesing B, Garcia R, Walding A, DeMajo R, et al. How teacher behaviors and perceptions, air change rates, and portable air purifiers affect indoor air quality in naturally ventilated schools. Front Public Health. 2024 Oct 3;12. doi:10.3389/fpubh.2024.1427116

8.	Martenies SE, Batterman SA. Effectiveness of using enhanced filters in schools and homes to reduce indoor exposures to PM2. 5 from outdoor sources and subsequent health benefits for children with asthma. Environ Sci Technol. 2018;52(18):10767–76.
